# Selenium Nucleophilicity and Electrophilicity in the Intra‐ and Intermolecular S_N_2 Reactions of Selenenyl Sulfide Probes

**DOI:** 10.1002/chem.202404580

**Published:** 2025-02-03

**Authors:** Andrea Madabeni, Lukas Zeisel, Oliver Thorn‐Seshold, Laura Orian

**Affiliations:** ^1^ Dipartimento di Scienze Chimiche Università degli Studi di Padova Via Marzolo 1 35129 Padova Italy; ^2^ Faculty of Chemistry and Food Chemistry, TU Dresden Bergstrasse 66 01069 Dresden Germany

## Abstract

Chalcogenide exchange reactions are an important class of bimolecular nucleophilic substitution reactions (S_N_2) involving sulfur and selenium species as nucleophile, central atom, and/or leaving group, which are fundamental throughout redox biology and metabolism. While thiol‐disulfide exchange reactions have been deeply investigated, those involving selenium are less understood, especially with regards to the polarised selenenyl sulfides RSe–SR’. This functional group, which is fundamental in the biochemistry of glutathione peroxidase and thioredoxin reductase enzymes, was recently incorporated in the molecular scaffold of a TrxR1 specific probe, “RX1”. Here, we investigate the S_N_2@S and S_N_2@Se reactions of selenenyl sulfides *in silico* to provide the first comprehensive overview of their kinetic and thermodynamic trends, referencing against symmetrical disulfides and diselenides. Then, the role of S_N_2@S and S_N_2@Se reactions in RX1 chemistry is explored, and a mechanistic picture of its biological chemistry is provided. Additionally, we quantify the role of alternative exchange reactions in the double‐exchange chemistry of RX1. This analysis rationalises the origins of RX1’s TrxR‐specificity even within thiol‐rich cellular environments and can support the design and applications of a range of selenenyl sulfide‐based bioactive probes. Particularly, we observe that the intramolecular S_N_2@Se reaction which restores RX1 ground state is an effective protective mechanism against unspecific activation by thiols, explaining its capacity to work in high‐thiol concentration.

## Graphical abstract

Chalcogenol‐dichalcogenide exchange reactions of linear and cyclic dichalcogenides are investigated in silico to gain insight into their relative rates. The unveiled role of double exchange reactions in the chemistry of RX1, a selenenyl sulfide TrxR1 probe, explains the origin of its specificity even under high‐thiol concentrations and provides hints to design new selective TrxR1 probes.

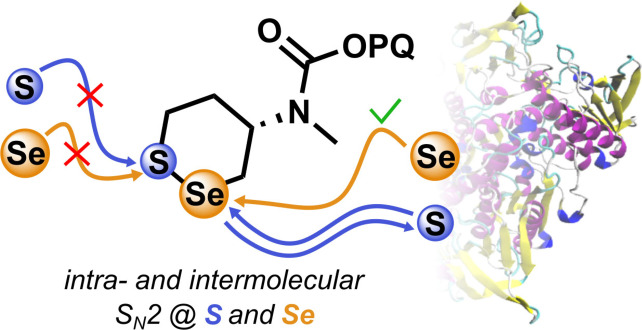

## 1 Introduction

**Scheme 1:**
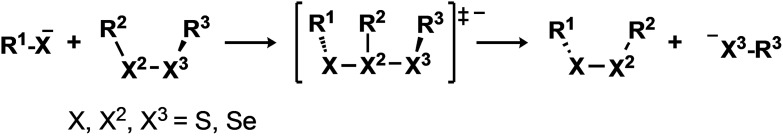
Chalcogenolate‐dichalcogenide exchange is concerted in solution.

Thiol‐disulfide exchange reactions are well‐known S_N_2 reactions, that proceed in a single concerted step in solution, with the incoming thiolate R^1^S^−^ attacking a disulfide sulfur (R^2^S−) to expel a thiolate R^3^S^−^ (Scheme 1, X=X^2^=X^3^=S).[^1^, ^2^, ^3^, ^4^, ^5^]

While disulfide (X^2^=X^3^=S) exchanges have been intensively studied, both experimentally and computationally, far less attention was devoted to the analogous reactions for selenenyl sulfides (X^2^=Se, X^3^=S): which are both computationally and experimentally more demanding.

Experiments on nucleophilic substitution at dichalcogenides involving Se started in the late 60s, with the discovery that thiols could reduce diselenides.[^6^, ^7^] Significant advances in nucleophilic substitutions @Se were made by Rabenstein and coworkers, who observed in 1989 that the symmetrical substitution of diselenides occurred 7 orders of magnitude faster than that of the corresponding disulfides.^[8]^ Nevertheless, the first near‐complete investigation comparing thiolates and selenolates as nucleophiles in reactions with dichalcogenides including selenenyl sulfides was published only in 2010 by Koppenol and coworkers.^[9]^ Among other results, they observed that selenium acts both as a better nucleophile and as a better electrophile than sulfur, although at neutral pH it did not provide any particular rate advantage over sulfur as a leaving group. While some of these observations had been made earlier,^[10]^ Koppenol's study provided the first systematic experimental perspective and it is still a reference point for research on this topic. Yet, knowledge gaps remained: e. g., that the rate of RSe^−^ attack @S in R^2^SeSR^3^ (a key rate needed to understand the selectivity of selenenyl sulfide reactivity in biology) was not described, since at the time there was no suitable synthetic methodology to access the unsymmetric selenenyl sulfides needed to measure it: which left this rate to guesswork.

**Figure 1:**
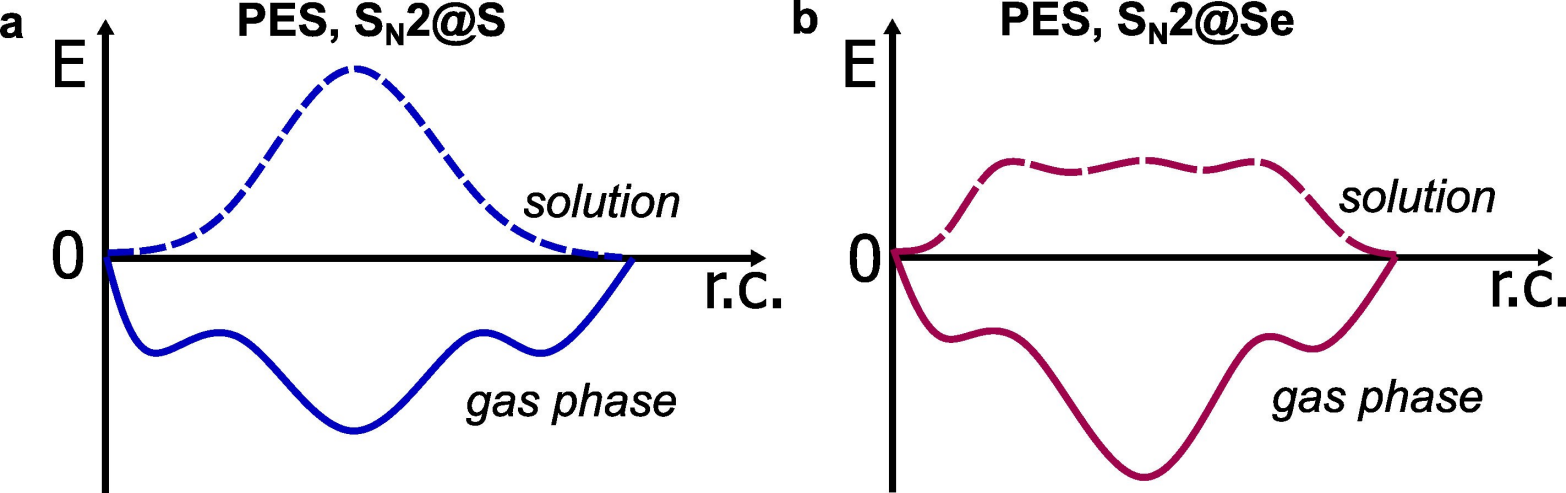
Schematic of the PES of thiolate attack (**a**) @S or (**b**) @Se of dichalcogenides, in gas phase (solid lines) or water (dashed lines) (Bortoli *et al*.;^[5]^ r.c.=reaction coordinate).

From the computational point of view, the history of nucleophilic substitutions at the selenium of dichalcogenides started in the 2000s. The seminal studies of Bachrach and coworkers[^11^, ^12^] probed gas phase mechanistic features of the potential energy surface (PES), comparing them to previous analyses on nucleophilic substitutions at sulfur,^[13]^ to provide evidence that the gas phase reactions occur by an addition‐elimination (A−E) mechanism passing through a stable S−X−X’ three‐center intermediate (TCI), regardless of whether the attack occurs at S or Se (Figure 1). However, in solution, nucleophilic substitutions of disulfides were found to occur as concerted S_N_2@S,^[13]^ while the situation remained unclear for substitutions at Se.

Bortoli *et al* tackled the S_N_2 vs A−E question in 2016, showing that for sulfur as the central atom, polar (implicit) solvation is enough to turn the gas phase addition‐elimination PES into a unimodal S_N_2 PES (Figure 1a). The results for selenium were more complex: the stable TCI that is key to its gas phase A−E substitution is less destabilised, transforming the PES into a broad plateau upon which the transition states and intermediates are located at similar energy values and geometries.^[5]^ Thus, while thiol‐disulfide exchanges are conventional S_N_2@S in solution, nucleophilic substitutions at selenium were predicted to occur in a transitional mechanistic regime in between A−E and S_N_2 extremes. The presence of an intermediate on the PES was also expected to be influenced by the nature of the substituent on the central Se atom, as previously observed for other nucleophilic substitutions at heavy atoms.^[4]^

A deep understanding of chalcogenol S_N_2 reactions @S and @Se is fundamentally needed to rationalise the reactivity of dichalcogenides in biology and medicine.^[15]^ Intra‐ and intermolecular selenenyl sulfides are kinetically‐activated intermediates in the fully reversible catalytic cycles of selenoenzymes such as glutathione peroxidase[^16^, ^17^, ^18^, ^19^] and thioredoxin reductase,[^20^, ^21^, ^22^] just like in organoselenium‐based glutathione peroxidase‐mimetic drugs such as ebselen.[^23^, ^24^, ^25^] Tuning the polarisation and reactivity of selenenyl sulfides has also been a topic of much interest for modifying their on‐cycle reaction rates and speciation.[^26^, ^27^, ^28^] Recently, following synthetic advances by Iwaoka[^21^, ^29^] and Thorn‐Seshold^[30]^ that unlocked access to unsymmetric aliphatic selenenyl sulfides, these have also been used as thiol‐ or selenol‐reactive groups with remarkable biological specificity patterns: e. g. as slowly‐reversible reagents for thiol‐mediated uptake,^[31]^ or as trapped‐reversible probes for enzyme activity.[^14^, ^32^, ^33^]

In this work we will focus on the selenenyl sulfide reactivity patterns underlying the bioactivity of RX1, a fluorogenic bioreduction probe based on a cyclic selenenyl sulfide, that is fully inert towards >1000‐fold challenge with monothiols yet rapidly reacts with the nanomolar‐concentration mammalian selenol‐thiol enzyme TrxR1, making it an excellent and selective reporter on the activity of TrxR1 in cells.^[14]^ Its background‐free fluorogenicity has enabled the first quantitative high‐throughput live‐cell screen for TrxR1 inhibitors, and the probe has found applications as a valuable tool to study redox biology.[^33^, ^34^, ^35^]

**Figure 2:**
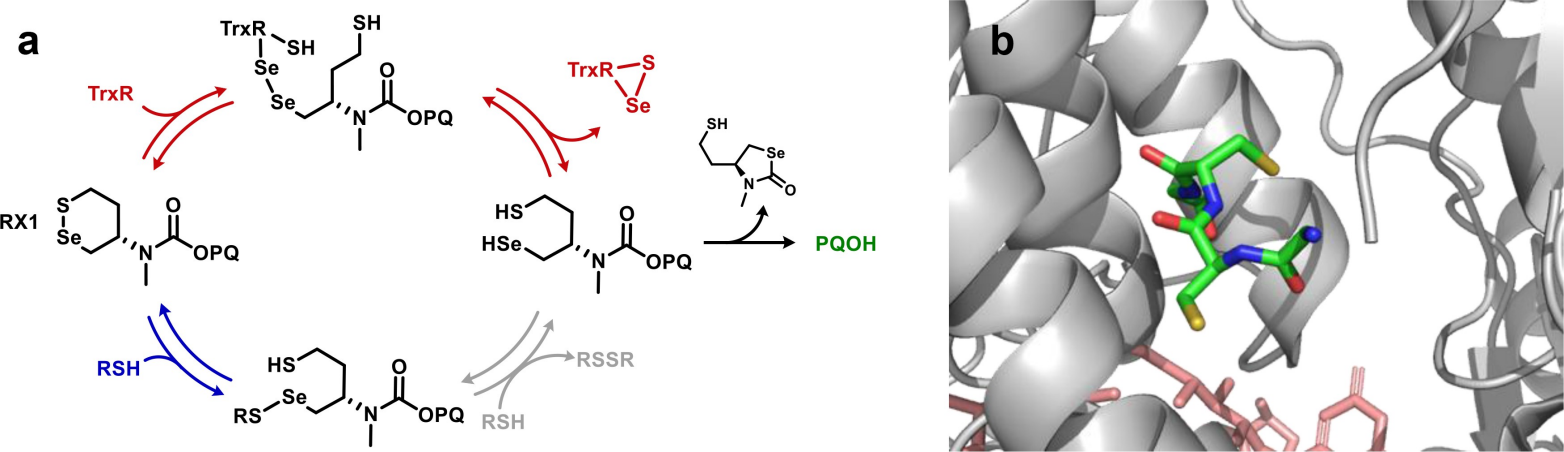
(**a**) Mechanism postulated for probe RX1 undergoing reduction (double exchange) by the enzyme TrxR (favored; red), or exchange with monothiols RSH (undesired; blue), from Zeisel *et al*.^[14]^ PQOH is the fluorogenic cargo. (**b**) The C‐terminal domain of mammalian TrxR1 ends with a Gly‐Cys‐Sec‐Gly‐CO_2_H motif (stick residues) that bears the double nucleophile (−SH/−SeH) for the double exchange (PDB: 1H6 V).

The selectivity of the RX1 probe was attributed to multiple kinetic effects arising from its electrophilic selenium atom. In brief, in the on‐target reaction pathway, the probe's fluorogenic cargo (PQOH) is released after double exchange with TrxR (nucleophilic substitutions first at probe Se, then at enzyme Se; Figure 2a, red pathway) that liberates the probe selenol, which rapidly performs *5‐exo‐trig* cyclization onto the carbonyl group to expel PQO^−^. The probe's selenenyl sulfide thus exploits both the enhanced electrophilicity of Se (in RX1) and the greater nucleophilicity of Se (in TrxR, Figure 2b) to ensure rapid on‐target double‐exchange with TrxR's unique selenolthiol.^[14]^ The off‐target reactivity of RX1 with cellular monothiols was however postulated to be *impeded* by its Se atom, due to the rational choice of selenenyl sulfide regiochemistry. This was argued since the first monothiol exchange intermediate RS−Se‐[RX1]‐SH is unlikely to undergo the required ‘productive’ intermolecular attack of a second external thiolate RSH *onto the S atom* of the S−Se bond, as it runs counter to the bond polarity (grey arrows). Instead, ‘counterproductive’ re‐closure by attack of the [RX1]‐SH thiol onto the Se atom, that expels the first monothiol RSH and reforms the original RX1 probe without signal generation, is much more likely: as this reaction is preorganised, intramolecular, and matches the S−Se polarity (blue pathway, Figure 2a). Taken together, these design features were postulated to grant RX1 its resistance towards monothiol‐triggered cargo release even at high thiol concentrations as are found in cells, while instead making it highly sensitive to TrxR.^[14]^

While the experimental performance of RX1 was convincing, its design logic had to make several kinetic assumptions that had never been tested experimentally nor computationally: e. g., the irrelevance of any [TrxR]Se^−^ attack @S in [RX1]RSeSR compared to its rate of attack @Se, or the significance of the intramolecular [RX1]‐SH re‐closure reaction, etc. Since these reactivity assumptions are crucial to understanding thiol‐type redox biology, and since the synthetic methods are now established to create compounds that can harness them, we decided that it would be timely to investigate these missing aspects of chalcogenolate‐dichalcogenide exchange reactions. The following work treats two systems: Section 2.1 covers a model system of monochalcogenols (MeXH nucleophile, EtX^2^X^3^Et dichalcogenide electrophile) that is studied for all combinations of X=S/Se, to provide a complete background for general rate and reactivity estimates; then, Section 2.2 applies this information to the specific chemical evolution pathways of the cyclic selenenyl sulfide RX1 system, to test the basis of its specificity for TrxR.

## Computational Methods

All DFT calculations were performed with the 2019.307 release of the Amsterdam Density Functional (ADF) software.[^36^, ^37^] The OLYP[^38^, ^39^] potential was used in combination with the TZ2P basis set with small frozen core approximation. Scalar relativistic effects were included by means of the Zeroth Order Regular Approximation (ZORA).^[40]^ This level of theory was previously benchmarked^[41]^ and applied[^5^, ^42^] to the study of the structure and reactivity of dichalcogenides and is recognized to be an affordable and accurate approach to tackle S_N_2 reactions.[^43^, ^44^, ^45^] All reactions were modelled using the solvent‐assisted proton‐exchange (SAPE) approach, with two water molecules to mediate proton transfers (see Paragraph 2). The Grimme D3 dispersion correction[^46^, ^47^] with the Becke‐Johnson damping factor[^48^, ^49^] was included to facilitate the assembling of an adduct with two water molecules for the solvent‐assisted mechanism. This level of theory is denoted as ZORA‐OLYP−D3(BJ)/TZ2P. Frequency calculations were carried out to assess the nature of each optimized geometry. All minima have only positive frequencies, while transition states have one imaginary frequency associated to the atomic motion connecting reactants to products. For selected reactions, an intrinsic reaction coordinate (IRC) profile has been computed to verify that the correct transition state was indeed located on the PES.^[50]^ For the model reaction (Section 2.1), single point calculations without dispersion (OLYP) were performed on top of OLYP−D3(BJ) optimized geometries (Table S1). As expected, removing dispersion led to increased activation energies, but only minor changes to reaction Gibbs free energies, and importantly, all trends were left unchanged.

Thermodynamic corrections were calculated by standard statistical thermodynamics relationships at 1 atm and 298.15 K under the perfect gas approximation. The effect of solvation (water) was included in a single‐point calculation on the gas phase optimized geometry employing the COSMO model of solvation as implemented in ADF, with the default parameters for dielectric constant (water), atomic radii and empirical scaling function.[^51^, ^52^] Standard state correction from gas phase to solution (1 bar to 1 M) was applied to all systems.^[53]^ All energies discussed in the main text are Gibbs free energies in water, unless specified otherwise. Molecular structures were illustrated using CYLview.^[54]^

For the RX1 system, the molecular scaffold was built starting from the available crystal structures of similar compounds.^[30]^ For intermediates which undergo further evolution (RX1‐SeSe and RX1‐SeS, see Section 2.2), a conformational search by the semiempirical GFN2‐xTB method of Grimme and coworkers^[55]^ combined with the CREST routine^[56]^ was carried out. The minimum located by this approach was then reoptimized at ZORA‐OLYP−D3(BJ)/TZ2P level of theory. Cartesian coordinates for all optimized structures are reported in Table S2.

## 2 Results and Discussion

First, results about the model linear dichalcogenides are presented (section 2.1); later, the discussion is extended to the cyclic dichalcogenide probe RX1 (section 2.2). In chalcogenol‐dichalcogenide exchange reactions, the deprotonated thiolate / selenolate anion is the active nucleophile^[9]^ which attacks the dichalcogenide bond (Scheme 1). At basic pH, when both thiols and selenols are completely deprotonated, thiolates are 1–2 orders of magnitude less nucleophilically reactive than selenolates.^[9]^ However, near neutral pH (at which biological exchanges occur), while selenols are expected to be ca. 99 % deprotonated, thiols are only <10 % deprotonated (pK_a_ of CysSH is ca. 8, pK_a_ of SecSeH is ca. 5),^[57]^ making selenol species 2–3 orders of magnitude more nucleophilically reactive than thiol species. To take into account the effects of chalcogenol acidity on nucleophilic reactivity, here we modelled all the nucleophilic substitutions at sulfur or selenium with a solvent‐assisted proton exchange (SAPE) mechanism, in which the nucleophile is considered to be protonated, but deprotonation occurs at the transition state *via* the mediation of explicit water molecules which drive the transfer of the proton from the nucleophile to the leaving group. This SAPE approach has been extensively applied to organochalcogen chemistry in the past fifteen years e. g. by Bayse[^58^, ^59^, ^60^] and Orian groups.[^61^, ^62^, ^63^] With SAPE, all substitutions occur in a concerted fashion,^[64]^ even for Se as the central atom in gas phase reactions:^[59]^ thus, no information about the existence of TCIs on the PES can be obtained. Since TCI species are expected to be extremely labile and almost isoenergetic with the nearby TSs, ignoring them should not affect any qualitative or quantitative insights into dichalcogenide exchange reactivity^[5]^ (further discussion in Supporting Note 1.)

### 2.1 Model Linear Dichalcogenide Reactivity

**Scheme 2:**
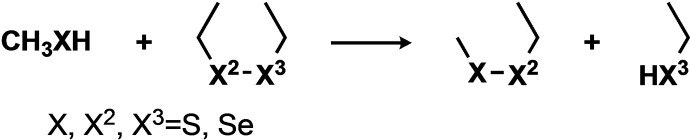
Model reaction for exchange between a chalcogenol (methyl) and a dichalcogenide (diethyl). SAPE Proton transfer from CH_3_XH to EtX^3^H is mediated by two water molecules (not shown).

We first studied an oversimplified system, reacting linear diethyl dichalcogenides (disulfides, diselenides, selenenyl sulfides) with methanethiol or methaneselenol nucleophiles (Scheme 2).

**Figure 3:**
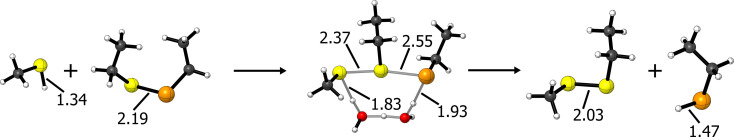
Optimized reactants, transition state and products (from left to right) for the reaction RSH + SSe. Relevant bond lengths are shown (in Å).

We computed energy landscapes for all eight possible reaction combinations of {X,X^2^,X^3^}as {S,Se}. As anticipated, all reactions occurred in a single step (concerted formation of the RX−X ^2^ bond with breaking of the X ^2^−X^3^ bond and SAPE proton transfer; Figure 3). Weakly bonded adducts (reactant complexes) can be located on the PES, but the solvation effect, and especially the inclusion of thermodynamics corrections, make these species destabilized with respect to the free reactants. Thus, all activation energies are given with respect to the free reactants.

We report Gibbs free energies of activation and of reaction for all eight reactions in Table 1 (to simplify notation, we represent e. g. thiol attack on the Se atom of a selenenyl sulfide as RSH + SeS, or thiol attack on the S atom of a selenenyl sulfide as RSH + SSe).

**Table 1:** Activation (ΔG^≠^) and reaction (ΔG_r_) energies (kcal mol^−1^) for the model reactions in Scheme 2.^a^

	MeXH + EtX ^2^X^3^Et	ΔG^≠^	ΔG_r_
1	RSH + SS	38.6	−1.6
2	RSeH + SS	34.0	−5.8
3	RSH + SSe	38.4	2.8
4	RSH+ SeS	32.5	−1.2
5	RSeH + SSe	33.4	−1.3
6	RSeH + SeS	27.9	−6.4
7	RSH + SeSe	33.2	4.0
8	RSeH + SeSe	28.0	−1.1

^**a**^ The dichalcogenide is written with the chalcogen at which the attack occurs in the first position, underlined.

The choice of selenium rather than sulfur as the nucleophile greatly affects activation energies ΔG^≠^ (ca. 4.5–5 kcal mol^−1^ lower) and reaction energies ΔG_r_ (ca. 4–5 kcal mol^−1^ more thermodynamically favorable), regardless of the choice of dichalcogenide and attack atom (e. g. entry 6 vs entry 4, Table 1), which matches the previous data trends reported of greater reactivity and product stability with selenol nucleophiles. By contrast, choosing a selenium leaving group rather than a sulfur leaving group barely affects ΔG^≠^ (e. g. entry 8 vs entry 6), although it raises ΔG_r_ (ca. 4–5 kcal mol^−1^ less thermodynamically favorable) again reflecting the greater stability of dichalcogenides with selenide rather than sulfide. Lastly, Se is a better electrophilic center than S: all reactions @Se have ΔG^≠^ 5–6 kcal mol^−1^ lower than the corresponding ones @S, e. g. with thiol attack at a selenenyl sulfide (entry 4 vs entry 3) having ΔΔG^≠^=6 kcal mol^−1^ in kinetic favour of attack @Se, which also gives the thermodynamically more stable products.

Noteworthily, these three effects can combine to give cases where the distinction between *kinetic* and *thermodynamic* aspects of reactivity becomes crucial: e. g., that thiol attack on a diselenide is kinetically favoured over its attack on a disulfide (by ca. 5.5 kcal mol^−1^), even though the diselenide attack is *thermodynamically* disfavoured (most positive reaction energy of all eight reactions studied). This matches many experimental observations that, in fully reversible equilibrations, diselenides accumulate as thermodynamically stable products,^[65]^ but underlines the “hidden” aspect that these apparently stable diselenides are also kinetically much faster to exchange. In the case of biological probes like RX1, where the probe is instead *irreversibly* triggered after *two sequential exchange steps*, it is the rates of each step that determine probe performance (not thermodynamic equilibria): thus kinetic discussion is crucial.

Taken together, these results provided a consistent picture of selenium‐directed reactivity being dominant both in terms of the nucleophilic atom, and the electrophilic attack site, that is consistent with the hypotheses underlying the RX1 mechanism‐based design. We now moved to model the RX1 reactions explicitly, by building in the cyclic selenenyl sulfide structure and the two‐step reactivity paths that it allows.

### 2.2 RX1 (Cyclic Selenenylsulfide) Reactivity

We now modelled thiol and disulfide exchanges with the cyclic Se−S bond in RX1 and with the intermediate adducts it forms after an initial exchange step^[14]^ (Figure 2). Once a selenol is generated on RX1 after productive double exchange, it will cyclize, irreversibly releasing the signal‐generating cargo. The specificity of RX1 for TrxR and its resistance against monothiols had thus been based on kinetic considerations underwriting the double exchange:

**§1** Selenoproteins like TrxR should react faster with RX1 than cysteine nucleophiles, as selenol nucleophiles attack dichalcogenides faster than thiols.

**§2** The selenenyl sulfide of RX1 directs all incoming chalcogenols to attack preferentially at Se, rather than at S: thus preventing direct selenol liberation (that could give direct cargo release) until an additional exchange on the intermediate is performed.

**§3** Thus, even when monothiols attack RX1 (at Se), cargo release would require a second, intermolecular S_N_2 in which another monothiol attacks *at the unfavored S end* of the newly‐formed selenenyl sulfide intermediate (whereas, any incoming monothiol would be instead expected to exchange again at Se, representing an unproductive cycling or “stalling” that blocks monothiol‐based cargo release), during its lifetime (that is predicted to be short, since intramolecular retro‐attack by the intermediate's thiol is *unusually* kinetically favoured due to preorganisation).

**§4** TrxR has a catalytic pocket with one Sec and one Cys residue that are pre‐organized in space to form an intramolecular CysS−SeSec bond.[^66^, ^67^] Thus, after [TrxR]SeH reacts with the probe, a second exchange reaction (intramolecular S_N_2) from [TrxR]SH is expected to occur at the [TrxR]Se atom of the [TrxR]Se−Se[RX1] intermediate, expelling [RX1]SeH: with the protein's spatial preorganisation helping to drive the exchange reaction at the correct, productive atom, while the symmetrical diselenide bond of the intermediate does not encourage reaction at the “wrong” selenium (in both respects, unlike the *intermolecular* reaction described in §3).

**Scheme 3:**
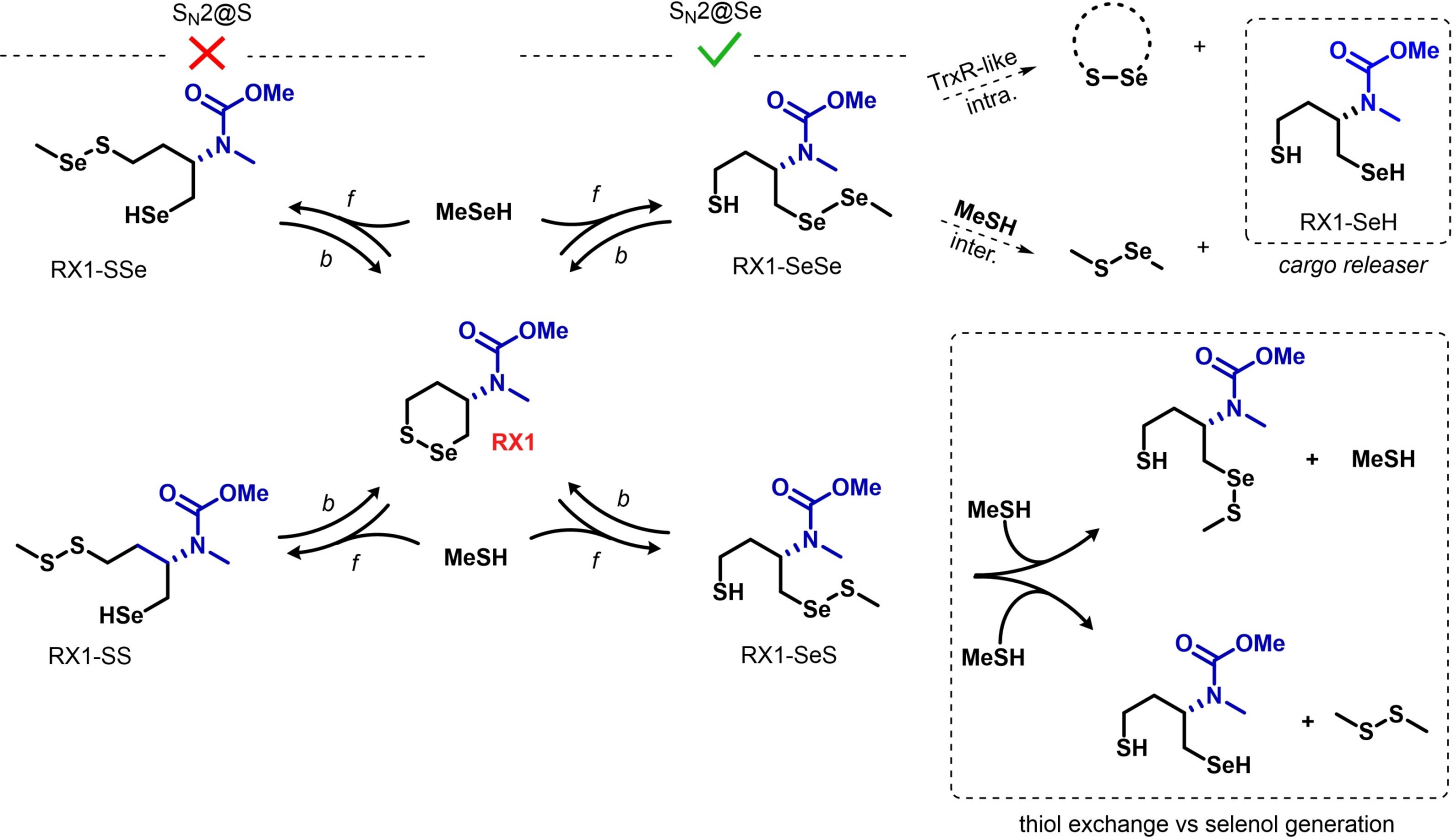
RX1 chemistry as modelled in this study. All processes are mediated by two water molecules (not represented). *f* stands for forward and *b* for backward process. The blue part was calculated either as shown (“RX1”) or else replaced by H for simplicity (“RX1‐H”).

The experimental performance of probe RX1 in selectively targeting TrxR1, as well as the non‐selectivity of its isoenergetic regioisomer probe “G1” that does not obey §2‐3, had supported this line of reasoning.^[14]^ We now used the SAPE approach to compute the dichalcogenide exchange reactions underlying these hypotheses, to discuss these four design points in a *quantitative* manner. Methanethiol and methaneselenol were used as simplified chalcogenol external nucleophiles; and RX1 was simplified by replacing both the fluorogenic cargo and the *N‐*substituent as methyl groups (Scheme 3).

Results for the first chalcogenol attack at RX1 are presented in Table 2. Very small qualitative differences can be seen between the reactivity of RX1 (5‐carbamate) and RX1‐H (5‐hydro), suggesting that the simplified system is an appropriate model for Se−S bond reactivity. Most of the conclusions drawn in Section 2.1 apply also to RX1 and RX1‐H reactivity.

**Table 2:** Activation energy and reaction energy (kcal mol^‐1^) for the forward (${\Delta G}_{F}^{\neq }$ ) and backward (${\Delta G}_{B}^{\neq }$ ) initial nucleophilic attack of MeSH or MeSeH onto RX1, or onto its simplified selenenyl sulfide core (RX1‐H).

		S_N_2@S		S_N_2@Se	
		MeSH	MeSeH	MeSH	MeSeH
RX1	${\Delta G}_{F}^{\neq }$	38.8	35.1	34.5	31.1
	${\Delta G}_{B}^{\neq }$	26.7	27.3	26.8	27.6
	ΔG_r_	12.2	7.8	7.7^a^	3.9^a^
RX1‐H	${\Delta G}_{F}^{\neq }$	39.1	34.0	33.6	28.9
	${\Delta G}_{B}^{\neq }$	27.1	26.3	26.4	26.7
	ΔG_r_	11.9	7.7	7.2	2.2

^a^ The ΔG_r_ of the process is computed with the product in a reactant‐like conformation, i. e., it is optimized in the structure in which it falls immediately after the transition state. Subsequent conformational optimisation leaves the reaction of RX1 @Se with thiols endergonic, while the corresponding reaction with selenols appears to be weakly exergonic (essentially isoergonic), with overall ΔG_r_ values of 3.86 and −1.18 kcal mol^−1^ respectively; but the energy differences due to product conformation do not affect trends or the overall discussion: see **Note 1**.

As expected, MeSeH is a better nucleophile than MeSH (3.5–5 kcal mol^−1^ lower ΔG^≠^), so selenoproteins can be considered preferential targets for RX1 (support for §1). The increased electrophilicity of Se also favours the S_N_2@Se attack site (ca. 4–5 kcal mol^−1^ lower ΔG^≠^) with either S or Se nucleophiles (support for §2, thus, Se electrophilicity disfavours the left part of Scheme 3 and so prevents direct cargo release). The combination of TrxR SeH nucleophilicity and RX1 Se electrophilicity results in an of 8 kcal mol^−1^ lower activation energy for the desired TrxR‐RX1[Se] reaction than the undesired RSH‐RX1[S] reaction. Also matching expectations, all forward reactions on the cyclic system are less thermodynamically favored than the analogous ones in Section 2.1, due to the entropic penalty of forming an addition intermediate. The most endergonic reaction, leading to RX1‐SS (the undesired thiol S_N_2@S reaction which would lead to non‐specific cargo release by selenol generation), has a ΔG_r_ of +12 kcal mol^−1^; while that for the desired TrxR‐SeSe‐RX1 is just +4 kcal mol^−1^ (Table 2).

**Figure 4:**
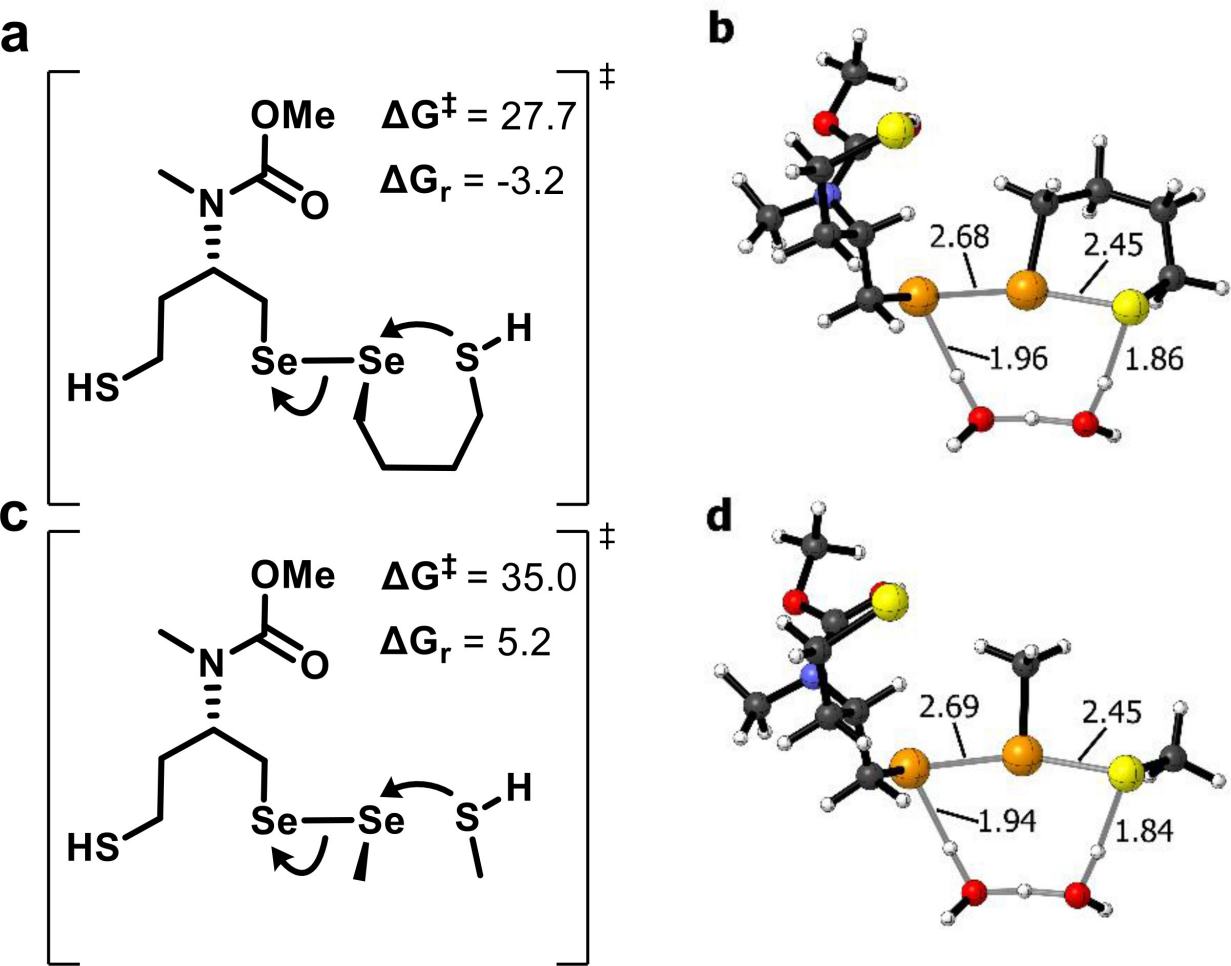
(**a**) Schematic representation of the transition state of the intramolecular, TrxR‐like step leading to selenol generation and (**c**) corresponding intermolecular process (water molecules are excluded for clarity). (**b**) Optimized transition state for the TrxR‐like process with explicit water molecules mediating the proton transfer from the thiol to the selenol moiety. (**d**) Corresponding optimized transition state for the intermolecular process. The TSs are reoriented (relative to the chemical structures) for optimal visualization. Energy values in kcal mol^−1^ and distances in Å.

After TrxR forms the desired intermediate (RX1‐SeSe in Scheme 3), the additional thiol of the protein should attack intramolecularly onto its Se, to expel the reduced RX1. This “resolution” step was computed for the simplified case of a 4‐mercaptobutan‐1‐selenyl motif bound to RX1[Se] (motif chosen as its possibility of forming a favoured 6‐membered ring was intended to capture some of the favourable preorganisation of the Cys and Sec residues in the protein) (Figure 4). Since this is an intramolecular attack of a thiol on Se in a dichalcogenide, we expected it to give an activation energy roughly comparable to the ${\Delta G}_{B}^{\neq }$ for MeSeH attacking RX1 @Se (27.2 kcal mo^−1^); and indeed, the onwards activation energy was computed as 27.7 kcal mol^−1^; and although this breaking of the diselenide bond is the thermodynamically most costly reaction step (Table 1), the intramolecular nature of the reaction with TrxR makes it thermodynamically favored (${\Delta G}_{r}$ =−3.2 kcal mol^−1^ for this model, with further discussion at Supporting Note 2; support for §4). By comparison, an *intermolecular* thiol‐on‐diselenide attack (Scheme 3, RX1‐SeSe evolution with MeSH; Figure 4c,d) would cost higher activation energy (35.0 kcal mol^−1^) and is thermodynamically unfavored (${\Delta G}_{r}$ =5.2 kcal mol^−1^), in line with the discussion in Section 2.1. Thus, while TrxR can reduce RX1 to RX1‐SeH by leveraging favorable energetics of ‐SeS‐ ring closure in the enzyme, other selenoproteins such as GPxs that lack a resolving Cys close to the Sec residue would not be able to hijack adventitious thiols like GSH to perform the resolution step. Taken together, these results from both initial and resolution steps now provide qualitative insights into the productive generation of RX1 selenol, driven by TrxR energetics and reaction molecularity.

It is equally important that reduction to the selenol should not occur with sequential monothiol exchanges. After initial thiol addition to RX1 gives the selenenyl sulfide intermediate, the selenol is only produced after a further S_N_2@S attack step. Matching Section 2.1, the activation energy for this unwanted resolution step (*productive intermolecular* monothiol S_N_2@S) is >40 kcal mol^−1^ (Table 3), whereas the *unproductive intermolecular* thiol exchange (stalling) is much more favoured (monothiol S_N_2@Se, ca. 34 kcal mol^−1^, Table 3): but now, these quantities can be compared to the even more favoured (and wanted) *counterproductive intramolecular* retro‐addition expelling the monothiol altogether and reforming RX1 (Table 2), occurring with an activation of 26.8 (from the preorganized product) or of 30.6 (if the product relaxes to the lowest conformer in energy). Thus, while the high cellular concentrations of monothiols will produce RX1‐SeS intermediate by initial monothiol exchange, the energetics of its follow‐up reaction options should prevent selenol generation (and undesired monothiol‐based cargo release) even in biological environments (support for §3). At our level of theory, we can therefore support the key result that *the restoration of the closed‐ring form of RX1 by retro‐addition is a more effective selectivity‐installing mechanism than the “thiol exchange stalling” reaction which the ‐SeS‐ polarisation in the RX1‐SeS intermediate causes*.

**Table 3:** Activation and reaction energies (kcal mol^−1^) for the thiol‐to‐RX1 addition intermediate (RX1‐SeS) to evolve by selenol generation (S_N_2@S) or thiol exchange (S_N_2@Se) (for carbamate RX1 and for simplified RX1‐H).

		Selenol generation	Thiol exchange
RX1	${\Delta G}^{\neq }$	40.3	34.5
	${\Delta G}_{r}$	4.2	0.1^a^
RX1‐H	${\Delta G}^{\neq }$	41.2	34.3
	${\Delta G}_{r}$	4.3	0.4^a^

^*a*^ *While the thiol exchange reaction rigorously has a* ${\Delta G}_{r}$ *=0, the energetics are reported with the products in the “reactant‐like” conformation, i. e., in the energy minimum immediately after the TS: thus, the conformer energies deviate slightly from the absolute minimum, but the overall discussion is not affected*.

## 3 Conclusions

This study offers a systematic perspective on chalcogenol‐dichalcogenide exchange reactions involving sulfur and selenium. Eight model reactions covering all combinations in which sulfur and selenium participate as nucleophilic chalcogenol, or as the central electrophilic atom and/or leaving group in the dichalcogenide, were considered. Our solvent‐assisted proton transfer results trends agree well with previous computational studies using fully deprotonated thiolate or selenolate as nucleophiles,^[5]^ and match to the experimental conclusions by Koppenol and coworkers for the reactions that were accessible at the time: i. e. selenols are better nucleophiles than thiols, the selenium atom in selenenyl sulfides is a better electrophilic center for attack than the sulfur, and, both selenols and thiols perform similarly as leaving groups.^[9]^

We then investigated the more complex reactivity that is biologically accessible to RX1, a selective probe for the selenol‐thiol enzyme TrxR1, that is based on a cyclic selenenyl sulfide which reversibly undergoes two sequential exchange reactions before performing an irreversible signal‐generating trapping step. The reaction steps with thiols and selenols matched trends from the linear dichalcogenide models, though with significantly directed reactivity due to the divergence between intramolecular (favored) and intermolecular (disfavored) reaction paths. By computing all the key on‐ and off‐target reactive steps of RX1, with thiols, selenols, and selenol‐thiols, we now provide a quantitative theoretical rationale for the postulates of Zeisel *et al* ^[14]^ that also delivers valuable, generally applicable information about selenenyl sulfide chemistry in the thiol‐rich biological environment.

Importantly, we could conclude that the intramolecular retro‐addition S_N_2@Se reaction of the monothiol‐attacked intermediate is the main mechanism protecting RX1 against non‐selective cargo release triggered by monothiols. Because of the previous lack of data, we had previously only been able to guess whether absolute retro‐addition rates were more, less, or equally important than their rates relative to those for exchange stalling (intermolecular exchange S_N_2@Se) in determining selectivity:^[14]^ yet this brings serious consequences for molecular design, since e. g. maximising the preorganisation of retro‐addition requires completely different structures than does maximising the polarisation of the SeS bond.

Now, by providing qualitative and quantitative data to estimate reactivity trends, this work will be able to direct the rational generation and tuning of new families of unsymmetric dichalcogenide reagents, not only for improved TrxR targeting, but also beyond. For example, we have already begun exploiting selenenyl sulfide‐based motifs to modulate thiol‐mediated cellular uptake (TMU), by rationally tuning the rates of their monothiol adduct intermediates’ intramolecular retro‐addition step.^[31]^ In brief, such tuning can e. g. employ proximal ammonium group H^+^ donors to perform the SAPE function experimentally, which accelerates forward addition, while also stabilising the adduct in a nonproductive H‐bonded conformation, which slows retro‐addition (“remote stabilisation” of the adduct); or conversely, can employ proximal carboxylates to repel the intermediate thiolate, destabilising ring opening (“remote destabilisation”).^[31]^ In TMU settings, the rates of initial thiol exchange, the lifetimes of the adducts before retro‐addition, and the kinetic lability of the intermediate towards intermolecular S_N_2@Se exchanges that drive the TMU process, are even more crucial to cellular performance than they are for RX1 (since TMU requires fully‐reversible thiol additivity without an irreversible trapping step): thus, exchange dynamics, of the type that we have calculated here, will continuously determine biological outcomes. Future studies in the TMU context will be far more complex in terms of the range of protein/transmembrane/aqueous environments, as well as the conformational and geometric factors dictating the accessibility of remote groups to the incipient thiolate.

More broadly, by providing a comprehensive and experimentally‐crosschecked perspective on S_N_2@Se and @S, this study now fully complements previous seminal mechanistic investigations on selenenyl sulfide exchange reactions,[^5^, ^11^] and the methodology used here can in principle be extended to diverse other settings for which the quantitative, physical, mechanistic picture of dichalcogenides’ biological chemistry will help in the design and use of powerful new dichalcogenide reagents.

## 4 Notes

The authors declare no competing financial interests.

## 5 End Note

**Note 1**: The ΔG_r_ of the process is computed with the product in a reactant‐like conformation, i. e., it is optimized in the structure in which it falls immediately after the transition state. In Table 2, for MeSH and MeSeH reaction @Se, since the adduct undergoes further evolution, a conformational search has been conducted (see computational methods). The absolute minima identified are 3.84 kcal mol^‐1^ and 5.10 kcal mol^‐1^ more stable for MeSH and MeSeH respectively. Thus, the reaction of RX1 with thiols remains endergonic, while the corresponding reaction with selenols appears to be weakly exergonic (essentially isoergonic), with an overall ΔG_r_ of 3.86 and −1.18 kcal mol^−1^ respectively; but the energy differences due to product conformation do not affect trends or the overall discussion.

## Supporting Information

PDF containing Supporting Notes 1–2 (extended discussion) and Tables S1–S2 (additionally reaction and activation energies, cartesian coordinates and electronic energies).

## Author Contributions

A.M. and L.O. set up the computational protocol. A.M. performed all calculations and wrote the first draft manuscript. L.O., L.Z. and O.T.‐S. edited the first draft manuscript. All authors participated in study design, results evaluation and interpretation, and edited and approved the final manuscript.

## Conflict of Interests

The authors declare no conflict of interest.

6

## Supporting information

As a service to our authors and readers, this journal provides supporting information supplied by the authors. Such materials are peer reviewed and may be re‐organized for online delivery, but are not copy‐edited or typeset. Technical support issues arising from supporting information (other than missing files) should be addressed to the authors.

Supporting Information

## Data Availability

The data that support the findings of this study are available in the supplementary material of this article.

## References

[1] S. M. Bachrach , D. C. Mulhearn , J. Phys. Chem. 1996, 100, 3535–3540.

[2] G. M. Whitesides , J. Houk , M. A. K. Patterson , J. Org. Chem. 1983, 48, 112–115.

[3] R. P. Szajewski , G. M. Whitesides , J. Am. Chem. Soc. 1980, 102, 2011–2026.

[4] T. A. Hamlin , M. Swart , F. M. Bickelhaupt , ChemPhysChem 2018, 19, 1315–1330. 29542853 10.1002/cphc.201701363 PMC6001448

[5] M. Bortoli , L. P. Wolters , L. Orian , F. M. Bickelhaupt , J. Chem. Theory Comput. 2016, 12, 2752–2761. 27096625 10.1021/acs.jctc.6b00253

[6] R. C. Dickson , A. L. Tappel , Arch. Biochem. Biophys. 1969, 130, 547–550. 5778666 10.1016/0003-9861(69)90068-x

[7] W. H. H. Guenther , J. Org. Chem. 1967, 32, 3931–3933.

[8] J. C. Pleasants , W. Guo , D. L. Rabenstein , J. Am. Chem. Soc. 1989, 111, 6553–6558.

[9] D. Steinmann , T. Nauser , W. H. Koppenol , J. Org. Chem. 2010, 75, 6696–6699. 20806911 10.1021/jo1011569

[10] R. Singh , L. Kats , Anal. Biochem. 1995, 232, 86–91. 8600838 10.1006/abio.1995.9956

[11] S. M. Bachrach , D. W. Demoin , M. Luk , J. V. Miller , J. Phys. Chem. A 2004, 108, 4040–4046.

[12] S. M. Bachrach , C. J. Walker , F. Lee , S. Royce , J. Org. Chem. 2007, 72, 5174–5182. 17550293 10.1021/jo070578s

[13] J. M. Hayes , S. M. Bachrach , J. Phys. Chem. A 2003, 107, 7952–7961.

[14] L. Zeisel , J. G. Felber , K. C. Scholzen , L. Poczka , D. Cheff , M. S. Maier , Q. Cheng , M. Shen , M. D. Hall , E. S. J. Arnér , J. Thorn-Seshold , O. Thorn-Seshold , Chem 2022, 8, 1493–1517. 35936029 10.1016/j.chempr.2022.03.010 PMC9351623

[15] A. Hamsath , M. Xian , Antioxid. Redox Signaling 2020, 33, 1143–1157. 10.1089/ars.2020.8083 PMC7698873 32151152

[16] L. Orian , P. Mauri , A. Roveri , S. Toppo , L. Benazzi , V. Bosello-Travain , A. De Palma , M. Maiorino , G. Miotto , M. Zaccarin , A. Polimeno , L. Flohé , F. Ursini , Free Radical Biol. Med. 2015, 87, 1–14. 26163004 10.1016/j.freeradbiomed.2015.06.011

[17] R. Masuda , R. Kimura , T. Karasaki , S. Sase , K. Goto , J. Am. Chem. Soc. 2021, 143, 6345–6350. 33887135 10.1021/jacs.1c02383

[18] L. Flohé , E. Schaich , W. Voelter , A. Wendel , Hoppe-Seyler's Z. Physiol. Chem. 1971, 352, 170–180. 5549563

[19] L. Flohé , S. Toppo , L. Orian , Free Radical Biol. Med. 2022, 187, 113–122. 35580774 10.1016/j.freeradbiomed.2022.05.003

[20] A. P. Lothrop , G. W. Snider , E. L. Ruggles , A. S. Patel , W. J. Lees , R. J. Hondal , Biochemistry 2014, 53, 654–663. 24422500 10.1021/bi400658g PMC3957198

[21] K. Arai , T. Matsunaga , H. Ueno , N. Akahoshi , Y. Sato , G. Chakrabarty , G. Mugesh , M. Iwaoka , Chem. A Eur. J. 2019, 25, 12751–12760. 10.1002/chem.201902230 31390113

[22] G. W. Snider , E. Ruggles , N. Khan , R. J. Hondal , Biochemistry 2013, 52, 5472–5481. 23865454 10.1021/bi400462j PMC3760785

[23] L. Orian , S. Toppo , Free Radical Biol. Med. 2014, 66, 65–74. 23499840 10.1016/j.freeradbiomed.2013.03.006

[24] K. P. Bhabak , G. Mugesh , Acc. Chem. Res. 2010, 43, 1408–1419. 20690615 10.1021/ar100059g

[25] A. Madabeni , M. Bortoli , P. A. Nogara , G. Ribaudo , M. Dalla Tiezza , L. Flohé , J. B. T. Rocha , L. Orian , Chem. A Eur. J. 2024, DOI 10.1002/chem.202403003. PMC11639659 39304519

[26] B. K. Sarma , G. Mugesh , J. Am. Chem. Soc. 2005, 127, 11477–11485. 16089478 10.1021/ja052794t

[27] K. P. Bhabak , G. Mugesh , Chem. A Eur. J. 2008, 14, 8640–8651. 10.1002/chem.200800963 18668498

[28] D. Bhowmick , G. Mugesh , Org. Biomol. Chem. 2015, 13, 10262–10272. 26372527 10.1039/c5ob01665g

[29] K. Arai , Y. Sato , I. Nakajima , M. Saito , M. Sasaki , A. Kanamori , M. Iwaoka , Bioorg. Med. Chem. 2021, 29, 115866. 33203607 10.1016/j.bmc.2020.115866

[30] L. Zeisel , M. S. Maier , O. Thorn-Seshold , Synthesis (Stuttg). 2023, 55, 1385–1393.

[31] F. Coelho , L. Zeisel , O. Thorn-Seshold , S. Matile , ChemistryEurope 2024, 2, DOI 10.1002/ceur.202400032.

[32] J. G. Felber , A. Kitowski , L. Zeisel , M. S. Maier , C. Heise , J. Thorn-Seshold , O. Thorn-Seshold , ACS Cent. Sci. 2023, 9, 763–776. 37122469 10.1021/acscentsci.2c01465 PMC10141580

[33] Z. Song , C. Fan , J. Zhao , L. Wang , D. Duan , T. Shen , X. Li , Biosensors 2023, 13, 811. 37622897 10.3390/bios13080811 PMC10452626

[34] R. J. Hondal , Redox Biol. 2022, 54, 102376. 35777199 10.1016/j.redox.2022.102376 PMC9253492

[35] D. M. Cheff , C. Huang , K. C. Scholzen , R. Gencheva , M. H. Ronzetti , Q. Cheng , M. D. Hall , E. S. J. Arnér , Redox Biol. 2023, 62, 102703. 37087975 10.1016/j.redox.2023.102703 PMC10149367

[36] ADF2019, SCM, Theoretical Chemistry, Vrije Universiteit, Amsterdam, The Netherlands, https://www.scm.com.

[37] G. te Velde , F. M. Bickelhaupt , E. J. Baerends , C. Fonseca Guerra , S. J. A. van Gisbergen , J. G. Snijders , T. Ziegler , J. Comput. Chem. 2001, 22, 931–967.

[38] N. C. Handy , A. J. Cohen , Mol. Phys. 2001, 99, 403–412.

[39] C. Lee , W. Yang , R. G. Parr , Phys. Rev. B 1988, 37, 785–789. 10.1103/physrevb.37.785 9944570

[40] E. Van Lenthe , E. J. Baerends , J. G. Snijders , J. Chem. Phys. 1994, 101, 9783–9792.

[41] F. Zaccaria , L. P. Wolters , C. Fonseca Guerra , L. Orian , J. Comput. Chem. 2016, 37, 1672–1680. 27093091 10.1002/jcc.24383

[42] M. Bortoli , F. Zaccaria , M. Dalla Tiezza , M. Bruschi , C. F. Guerra , F. Matthias Bickelhaupt , L. Orian , Phys. Chem. Chem. Phys. 2018, 20, 20874–20885. 30066704 10.1039/c8cp02748j

[43] A. P. Bento , M. Solà , F. M. Bickelhaupt , J. Comput. Chem. 2005, 26, 1497–1504. 16092145 10.1002/jcc.20261

[44] A. P. Bento , F. M. Bickelhaupt , J. Org. Chem. 2007, 72, 2201–2207. 17300206 10.1021/jo070076e

[45] P. Vermeeren , T. Hansen , P. Jansen , M. Swart , T. A. Hamlin , F. M. Bickelhaupt , Chem. A Eur. J. 2020, 26, 15538–15548. 10.1002/chem.202003831 PMC7756690 32866336

[46] S. Grimme , J. Antony , T. Schwabe , C. Mück-Lichtenfeld , Org. Biomol. Chem. 2007, 5, 741–758. 17315059 10.1039/b615319b

[47] S. Grimme , Wiley Interdiscip. Rev.: Comput. Mol. Sci. 2011, 1, 211–228.

[48] A. D. Becke , E. R. Johnson , J. Chem. Phys. 2005, 123, 154101. 16252936 10.1063/1.2065267

[49] E. R. Johnson , I. D. Mackie , G. A. DiLabio , J. Phys. Org. Chem. 2009, 22, 1127–1135.

[50] L. Deng , T. Ziegler , Int. J. Quantum Chem. 1994, 52, 731–765.

[51] A. Klamt , G. Schüürmann , J. Chem. Soc. Perkin Trans. 2 1993, 799–805.

[52] C. C. Pye , T. Ziegler , Theor. Chem. Acc. 1999, 101, 396–408.

[53] J. Ho , A. Klamt , M. L. Coote , J. Phys. Chem. A 2010, 114, 13442–13444. 21133342 10.1021/jp107136j

[54] Legault, C. Y. CYLview1.0b; Université de Sherbrooke: Sherbrooke, QC, Canada, 2020. http://www.cylview.org.

[55] S. Grimme , C. Bannwarth , P. Shushkov , J. Chem. Theory Comput. 2017, 13, 1989–2009. 28418654 10.1021/acs.jctc.7b00118

[56] P. Pracht , F. Bohle , S. Grimme , Phys. Chem. Chem. Phys. 2020, 22, 7169–7192. 32073075 10.1039/c9cp06869d

[57] R. E. Huber , R. S. Criddle , Arch. Biochem. Biophys. 1967, 122, 164–173. 6076213 10.1016/0003-9861(67)90136-1

[58] C. A. Bayse , K. N. Ortwine , Eur. J. Inorg. Chem. 2013, 21, 3680–3688.

[59] S. Antony , C. A. Bayse , Inorg. Chem. 2011, 50, 12075–12084. 22059718 10.1021/ic201603v

[60] C. A. Bayse , J. Phys. Chem. A 2007, 111, 9070–9075. 17718544 10.1021/jp072297u

[61] A. Madabeni , P. A. Nogara , M. Bortoli , J. B. T. Rocha , L. Orian , Inorg. Chem. 2021, 60, 4646–4656. 33587617 10.1021/acs.inorgchem.0c03619 PMC8763373

[62] R. Masuda , S. Kuwano , S. Sase , M. Bortoli , A. Madabeni , L. Orian , K. Goto , Bull. Chem. Soc. Jpn. 2022, 95, 1360–1379.

[63] A. Madabeni , L. Orian , Int. J. Mol. Sci. 2023, 24(9), 7754. 10.3390/ijms24097754 PMC10178455 37175462

[64] C. A. Bayse , Org. Biomol. Chem. 2011, 9, 4748–4751. 21597638 10.1039/c1ob05497j

[65] L. A. Sancineto , F. Mangiavacchi , A. Dabrowska , A. J. Pacuła-Miszewska , M. Obieziurska-Fabisiak , C. Scimmi , V. Ceccucci , J. Kong , Y. Zhao , G. Ciancaleoni , V. Nascimento , B. Rizzuti , M. Bortoli , L. Orian , A. Kula-Pacurar , H. Yang , J. Ścianowski , Y. Lei , K. Pyrc , C. Santi , Sci. Rep. 2024, 14, 24751, 10.1038/s41598-024-75519-6. 39433805 PMC11494035

[66] H. J. Reich , R. J. Hondal , ACS Chem. Biol. 2016, 11, 821–841. 26949981 10.1021/acschembio.6b00031

[67] R. J. Hondal , E. L. Ruggles , Amino Acids 2011, 41, 73–89. 20397034 10.1007/s00726-010-0494-6 PMC2935959

