# Supplementary material for: Selenium Nucleophilicity and Electrophilicity in the Intra‐ and Intermolecular SN2 Reactions of Selenenyl Sulfide Probes
Source: Chemistry. 2025 Feb 3;31(12):e202404580. doi: 10.1002/chem.202404580 (PMC11855254; doi:10.1002/chem.202404580)
Supplement: Supplementary file 1 — Supporting Information [file CHEM-31-e202404580-s001.pdf]

Chemistry–A European Journal

Supporting Information

Selenium Nucleophilicity and Electrophilicity in the Intra- and Intermolecular S_N2 Reactions of Selenenyl Sulfide Probes

Andrea Madabeni, Lukas Zeisel, Oliver Thorn-Seshold, and Laura Orian*

Supporting Information

Selenium Nucleophilicity and Electrophilicity in the Intra- and Intermolecular S_N2 Reactions of Selenenyl Sulfide Probes

Andrea Madabeni[a], Lukas Zeisel[b], Oliver Thorn-Seshold[b], Laura Orian[a]*

[a] Dipartimento di Scienze Chimiche Università degli Studi di Padova Via Marzolo 1 35129 Padova, Italy

[b] Faculty of Chemistry and Food Chemistry, TU Dresden, Bergstrasse 66, 01069 Dresden, Germany

Table of Contents

Table S1 Activation (ΔG ‡) and reaction (ΔGr) energies (kcal mol^{-1}) for the eight model reactions in Scheme 2 (main text). Level of theory ZORA-OLYP/TZ2P // ZORA-OLYP-D3(BJ)/TZ2P. S1

Table S2. Cartesian coordinates (Å), imaginary frequencies Nimag (cm^{-1}), and energies E (kcal mol^{-1}) for all structures investigated in this work. Level of theory: ZORA-OLYP-D3(BJ)/TZ2P. S2

Supporting Note 1: It must be kept in mind that while selenols are usually completely deprotonated at neutral pH,[1] thiols are mostly in equilibrium between their protonated and deprotonated form, and this might affect the stabilization of reactants and products based on the way in which the solvent stabilizes the charged species. For the reaction types under investigation, *qualitatively* similar trends in results were obtained by Bortoli *et al* when considering both the selenolate and the thiolate as completely deprotonated.[2] For example, their comparison of SeH+SeS vs SeH + SeSe provides the same qualitative (tiny) effect of the leaving group as we find; even if Bortoli *et al* predicted the selenolate to be a better leaving group than the thiolate, from a thermodynamic point of view, in the RSH+S̲X reaction (X=S, Se) (though the energy difference between the two reactions was only 0.2 kcal mol^{-1}, both reactions appearing to be essentially thermoneutral).

Supporting Note 2: It should be mentioned that this ΔG_r must be considered with particular caution, because a six-membered ring is formed in our model reaction: the situation will clearly be different in TrxR, where if the two consecutive Cys and Sec residues interact with RX1, an eight-membered ring is formed, whose structure and stability can be expected to be influenced by the protein backbone constraints. Indeed, the formation of this cyclic intermediate is an integral part of the canonical catalytic mechanism of TrxR. Thus, the same reaction in TrxR might be even more favored than in the simplified model. Nevertheless, our results provide at least a qualitative insight into the productive selenol generation step of RX1 mechanism through intramolecular ring closure.

Table S1 Activation ($\Delta G^{\ddagger}$) and reaction (ΔG_r) energies (kcal mol^{-1}) for the eight model reactions in Scheme 2 (main text). Level of theory ZORA-OLYP/TZ2P // ZORA-OLYP-D3(BJ)/TZ2P.

		$\Delta G^{\ddagger}$	ΔG_r
1	SH + SS	65.27	-3.43
2	SeH + SS	60.27	-7.82
3	SH + SSe	64.71	1.08
4	SH+ SeS	58.78	-3.27
5	SeH + SSe	59.21	-3.30
6	SeH + SeS	53.96	-8.66
7	SH + SeSe	58.49	2.04
8	SeH + SeSe	53.09	-3.35

Table S2. Cartesian coordinates (Å), imaginary frequency Nimag (cm^{-1}), energies E (kcal mol^{-1}), for all structures investigated in this work. Level of theory: ZORA-OLYP-D3(BJ)/TZ2P.

Minimal Model

SS

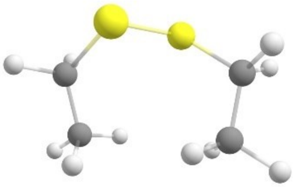

E= -1849.10
Nimag=0

C	0.052572000	0.668795000	-1.577666000
C	-0.555737000	-0.064093000	-0.404018000
C	0.668732000	-3.323860000	-0.942904000
C	1.171839000	-3.211343000	-2.363804000
H	0.508518000	1.615225000	-1.266735000
H	-0.699351000	0.898272000	-2.335426000
H	-1.359528000	0.536243000	0.034506000
H	0.191536000	-0.261690000	0.366877000
H	-0.420755000	-3.386860000	-0.914840000
H	0.843420000	-4.060043000	-2.973842000
H	2.262866000	-3.183558000	-2.397582000
S	1.433109000	-0.171856000	-2.422149000
S	0.564042000	-1.782805000	-3.320795000
H	1.082656000	-4.220743000	-0.470616000
H	-0.977827000	-1.013801000	-0.730932000
H	0.982093000	-2.456505000	-0.362801000

SSe

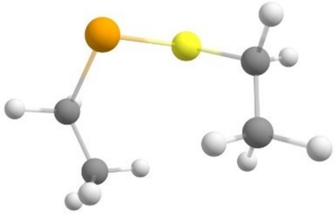

Nimag=0
E= -1831.31

C	0.052277000	0.728040000	-1.552905000
C	-0.575202000	-0.053247000	-0.421124000
C	0.696466000	-3.345419000	-0.931162000
C	1.178831000	-3.296813000	-2.359893000
H	0.510783000	1.655532000	-1.192259000
H	-0.688764000	0.999358000	-2.307230000
H	-1.390448000	0.524748000	0.026217000
H	0.159137000	-0.278880000	0.354354000
H	-0.391561000	-3.420959000	-0.882258000
H	0.844213000	-4.162676000	-2.939890000
H	2.265969000	-3.243194000	-2.422414000
S	1.438889000	-0.091886000	-2.409602000
Se	0.497873000	-1.782583000	-3.431071000
H	1.128592000	-4.212562000	-0.419343000
H	-0.984209000	-0.991391000	-0.796057000
H	1.005339000	-2.446690000	-0.398090000

SeSe

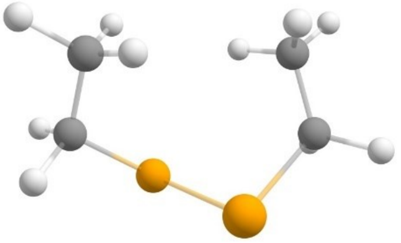

E= -1814.59
Nimag=0

C	0.030063000	0.794990000	-1.507670000
C	-0.600238000	-0.040777000	-0.421275000
C	0.722281000	-3.345141000	-0.934798000
C	1.166277000	-3.354886000	-2.376612000
H	0.475290000	1.715082000	-1.116512000
H	-0.687046000	1.064776000	-2.283064000
H	-1.444576000	0.496965000	0.024300000
H	0.118479000	-0.272822000	0.366914000
H	-0.365199000	-3.397324000	-0.855204000
H	0.787978000	-4.226036000	-2.920473000
H	2.252253000	-3.338771000	-2.469588000
Se	1.554880000	-0.054414000	-2.436972000
Se	0.496453000	-1.848133000	-3.467836000

H	1.152091000	-4.201693000	-0.403587000
H	-0.972507000	-0.977384000	-0.836692000
H	1.061708000	-2.433055000	-0.443659000

SH

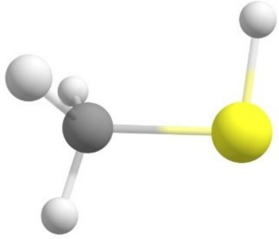

E= -626.90
Nimag=0

S	0.321682000	-4.734858000	-4.069697000
C	-0.612326000	-4.041416000	-5.464493000
H	-0.368391000	-2.989481000	-5.617803000
H	-0.303731000	-4.607933000	-6.344754000
H	-1.686128000	-4.167914000	-5.321256000
H	-0.190755000	-3.921529000	-3.128544000

SeH

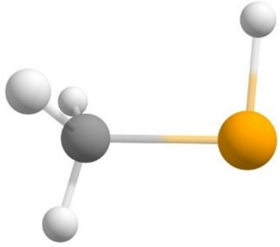

E= -604.71
Nimag=0

Se	0.489895000	-4.928663000	-4.173189000
C	-0.652936000	-4.043616000	-5.503293000
H	-0.434204000	-2.977642000	-5.535500000
H	-0.399785000	-4.500694000	-6.460269000
H	-1.702100000	-4.223949000	-5.275418000
H	-0.035152000	-4.147696000	-3.041283000

EtSH

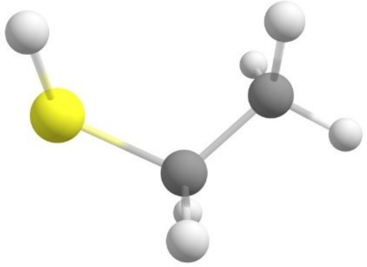

E= -1003.95
Nimag=0

C	-0.049621000	-3.756892000	-1.587911000
C	1.243266000	-3.340138000	-2.262394000
H	-0.703902000	-4.274891000	-2.293936000
H	1.791195000	-4.205390000	-2.641921000
H	1.899092000	-2.820541000	-1.558725000
S	1.016806000	-2.145911000	-3.617800000
H	0.159123000	-4.436169000	-0.754226000
H	-0.585074000	-2.885157000	-1.206415000
H	0.243602000	-2.943402000	-4.377677000

EtSeH

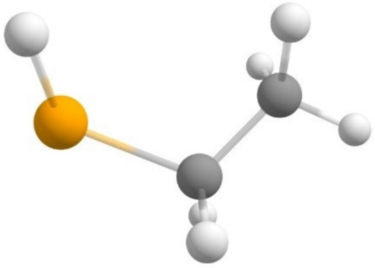

E= -981.53
Nimag=0

C	-0.048226000	-3.775687000	-1.571479000
C	1.252829000	-3.344947000	-2.213990000
H	-0.687911000	-4.288720000	-2.293733000
H	1.812041000	-4.190908000	-2.614469000
H	1.895800000	-2.818103000	-1.505217000
Se	1.017573000	-2.044046000	-3.675310000
H	0.150470000	-4.464909000	-0.742432000
H	-0.597101000	-2.914458000	-1.185322000
H	0.219012000	-2.966712000	-4.499051000

P-SSe

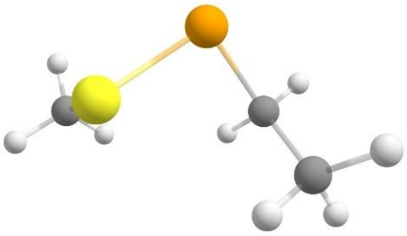

E= -1454.80
Nimag=0

C	-0.029807000	0.427190000	-1.688640000
C	0.243146000	0.247344000	-0.213414000
H	-0.368550000	1.436073000	-1.926882000
H	-0.772256000	-0.282978000	-2.065054000
H	-0.650738000	0.490780000	0.371578000
H	1.050954000	0.909967000	0.103010000
Se	1.551205000	0.073386000	-2.819378000
H	0.535737000	-0.781540000	0.006812000
S	2.873675000	1.656103000	-2.109752000
C	2.351034000	3.079444000	-3.107234000
H	3.003049000	3.909311000	-2.819093000
H	2.474438000	2.865317000	-4.168799000
H	1.315093000	3.341334000	-2.890160000

P-SS

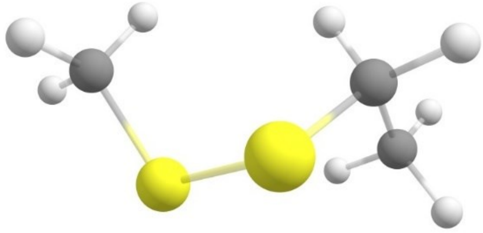

E= -1473.01
Nimag=0

C	0.033922000	0.472175000	-1.704606000
C	0.257326000	0.237898000	-0.225743000
H	-0.319259000	1.486343000	-1.903909000
H	-0.703239000	-0.224707000	-2.117473000

H	-0.658790000	0.447058000	0.335961000
H	1.045635000	0.897162000	0.142247000
S	1.514211000	0.176152000	-2.727354000
H	0.556953000	-0.795647000	-0.039490000
S	2.805204000	1.614616000	-2.101757000
C	2.320250000	3.046384000	-3.105225000
H	3.021475000	3.847437000	-2.854865000
H	2.397378000	2.804148000	-4.165335000
H	1.305915000	3.362712000	-2.859457000

P-SeS

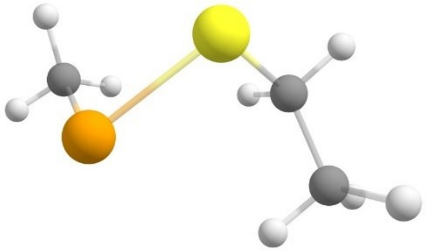

E= -1455.04
Nimag=0

C	0.023489000	0.455661000	-1.675962000
C	0.221593000	0.185196000	-0.199662000
H	-0.291507000	1.485133000	-1.858713000
H	-0.736021000	-0.206370000	-2.106087000
H	-0.696828000	0.404990000	0.354460000
H	1.020214000	0.818003000	0.192296000
S	1.497306000	0.122030000	-2.696437000
H	0.493513000	-0.859071000	-0.031547000
Se	2.914016000	1.639091000	-2.026325000
C	2.347425000	3.130229000	-3.173925000
H	3.034758000	3.954626000	-2.970099000
H	2.419647000	2.821795000	-4.215107000
H	1.329376000	3.420417000	-2.919899000

P-SeSe

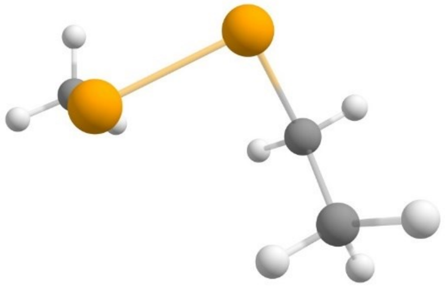

E= -1437.94
Nimag=0

C	-0.048196000	0.410098000	-1.656283000
C	0.208832000	0.188967000	-0.183980000
H	-0.354731000	1.433765000	-1.872967000
H	-0.810185000	-0.269172000	-2.050426000
H	-0.685797000	0.434089000	0.399361000
H	1.024935000	0.829753000	0.155896000
Se	1.523316000	0.030533000	-2.792683000
H	0.480973000	-0.850151000	0.013282000
Se	2.970035000	1.678399000	-2.040365000
C	2.384495000	3.169026000	-3.180578000
H	3.033768000	4.013741000	-2.938412000
H	2.500814000	2.886896000	-4.225045000
H	1.348722000	3.415787000	-2.954806000

Water
E= -324.93
Nimag=0

O	0.000000000	0.000000000	-1.815228000
H	0.000000000	0.760210000	-1.220499000
H	0.000000000	-0.760210000	-1.220499000

TS-SH+SS

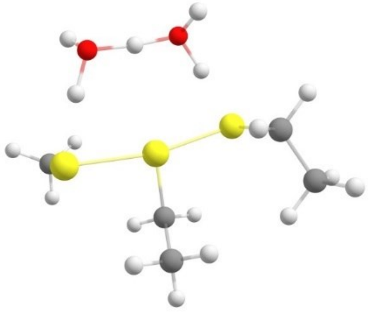

E= -3120.51
Nimag=-768

C	0.126575000	0.404266000	-1.724861000
C	0.212616000	0.197000000	-0.226725000
C	-0.028993000	-3.662717000	-1.457022000
C	1.168016000	-3.312350000	-2.322089000
H	-0.041088000	1.454310000	-1.966808000
H	-0.675567000	-0.183351000	-2.169566000
H	-0.719357000	0.516599000	0.252746000
H	1.034711000	0.781893000	0.188124000
H	-0.830159000	-4.092142000	-2.062923000
H	1.549306000	-4.207011000	-2.823611000
H	1.973551000	-2.885486000	-1.718571000
S	1.633751000	-0.108549000	-2.614509000
S	0.767474000	-2.089010000	-3.607858000
H	0.254127000	-4.390679000	-0.689095000
H	0.383095000	-0.853817000	0.010370000
H	-0.419320000	-2.773339000	-0.961118000
S	2.885802000	1.794558000	-1.893707000
C	2.268736000	2.982947000	-3.114275000
H	3.033727000	3.736200000	-3.316702000
H	2.011856000	2.453768000	-4.034510000
H	1.377625000	3.481811000	-2.729314000
H	3.963172000	0.893974000	-3.010172000
O	4.539466000	0.282991000	-3.723865000
H	4.726879000	0.855310000	-4.478225000
H	3.958512000	-0.705934000	-4.115827000
O	3.473385000	-1.720989000	-4.563057000
H	2.433047000	-1.904226000	-4.247226000
H	3.985043000	-2.474819000	-4.243900000

TS-SeH+SS

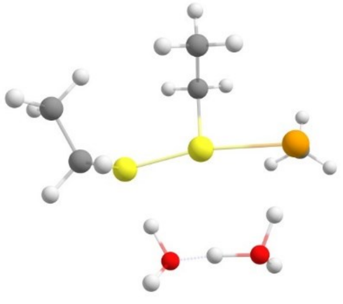

E= -3103.04
Nimag=-597

C	0.133873000	0.396483000	-1.706413000
C	0.166608000	0.193716000	-0.205734000
C	-0.037019000	-3.683275000	-1.456482000
C	1.158417000	-3.348257000	-2.329572000
H	-0.002266000	1.448838000	-1.957055000
H	-0.666788000	-0.176291000	-2.173218000
H	-0.775409000	0.530695000	0.241181000
H	0.983767000	0.767207000	0.234318000
H	-0.861355000	-4.067023000	-2.061959000
H	1.504284000	-4.240010000	-2.861226000
H	1.985481000	-2.963350000	-1.727095000
S	1.658419000	-0.151913000	-2.548054000
S	0.781771000	-2.080766000	-3.579278000
H	0.232215000	-4.442908000	-0.714670000
H	0.311855000	-0.858648000	0.041342000
H	-0.388455000	-2.796059000	-0.928922000
Se	2.990884000	1.883910000	-1.767200000
C	2.239245000	3.063663000	-3.145300000
H	3.002677000	3.765171000	-3.483278000
H	1.897145000	2.438077000	-3.970037000
H	1.398540000	3.614647000	-2.724562000
H	4.053458000	0.874772000	-3.027120000
O	4.584591000	0.272320000	-3.770639000
H	4.723744000	0.853698000	-4.529543000
H	3.999542000	-0.693467000	-4.129338000
O	3.493648000	-1.732684000	-4.572415000
H	2.474413000	-1.905883000	-4.250999000
H	4.002705000	-2.491457000	-4.261025000

TS-SH+SSe

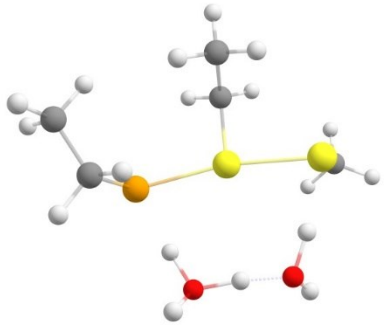

E= -3102.80
Nimag=-603

C	0.117614000	0.457163000	-1.729544000
C	0.235296000	0.134896000	-0.254279000
C	-0.061609000	-3.755338000	-1.402531000
C	1.140220000	-3.344313000	-2.228528000
H	-0.029607000	1.525897000	-1.888948000
H	-0.708612000	-0.078516000	-2.194934000
H	-0.675270000	0.439001000	0.274040000
H	1.082823000	0.666488000	0.181254000
H	-0.782230000	-4.307089000	-2.010655000
H	1.631717000	-4.216881000	-2.664411000
H	1.866157000	-2.786572000	-1.634666000
S	1.597145000	-0.018680000	-2.686562000
Se	0.651180000	-2.144896000	-3.716549000
H	0.247779000	-4.397211000	-0.569391000
H	0.383505000	-0.934856000	-0.104377000
H	-0.568867000	-2.881505000	-0.990729000
S	2.863512000	1.835559000	-1.938451000
C	2.286087000	3.059990000	-3.142631000
H	3.042961000	3.838559000	-3.264465000
H	2.097293000	2.568647000	-4.099209000
H	1.360855000	3.519572000	-2.790464000
H	3.979880000	0.913337000	-3.057123000
O	4.553482000	0.307815000	-3.746973000
H	4.748377000	0.874728000	-4.503467000
H	3.972141000	-0.715213000	-4.137012000
O	3.523371000	-1.722522000	-4.566524000
H	2.475143000	-1.902304000	-4.305507000
H	4.015648000	-2.464547000	-4.191658000

TS-SeH+SSe

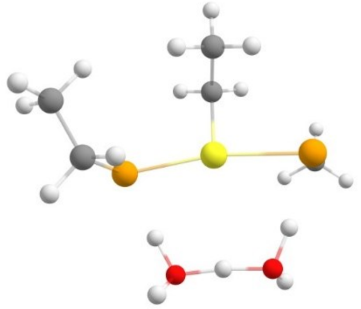

E= -3085.65
Nimag=-493

C	0.125261000	0.458475000	-1.711889000
C	0.181782000	0.138630000	-0.232724000
C	-0.077333000	-3.790070000	-1.415774000
C	1.125732000	-3.385621000	-2.242985000
H	0.014306000	1.530115000	-1.879404000
H	-0.699988000	-0.056117000	-2.202676000
H	-0.739280000	0.467049000	0.261826000
H	1.026044000	0.650837000	0.231787000
H	-0.820470000	-4.301296000	-2.032196000
H	1.590232000	-4.256252000	-2.711322000
H	1.871856000	-2.863849000	-1.641544000
S	1.624209000	-0.062564000	-2.617646000
Se	0.654885000	-2.129828000	-3.689869000
H	0.223907000	-4.467508000	-0.608200000
H	0.298362000	-0.933959000	-0.074363000
H	-0.554251000	-2.916293000	-0.969252000
Se	2.987262000	1.906135000	-1.798158000
C	2.287296000	3.152538000	-3.144740000
H	3.065505000	3.865108000	-3.420573000
H	1.972861000	2.569226000	-4.010082000
H	1.433360000	3.686257000	-2.728364000
H	4.071551000	0.885856000	-3.080568000
O	4.592705000	0.292725000	-3.813390000
H	4.719821000	0.870697000	-4.576924000
H	4.006382000	-0.708023000	-4.161574000
O	3.535412000	-1.740266000	-4.578411000
H	2.504367000	-1.905940000	-4.311010000
H	4.024214000	-2.484853000	-4.204270000

TS-SH+SeS

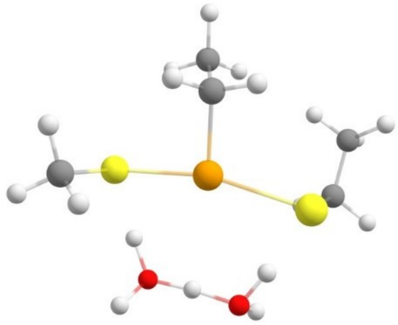

E= -3108.34
Nimag=-758

C	-0.056270000	0.910113000	-1.331767000
C	0.064410000	0.128045000	-0.038088000
C	0.125558000	-3.749359000	-1.558326000
C	1.083559000	-2.949982000	-2.412587000
H	0.034490000	1.984645000	-1.146509000
H	-1.026235000	0.720852000	-1.799259000
H	-0.718933000	0.426423000	0.666763000
H	1.037238000	0.300150000	0.428566000
H	-0.605244000	-4.264282000	-2.183623000
H	1.633756000	-3.586099000	-3.105904000
H	1.795522000	-2.375410000	-1.821279000
S	1.235363000	0.457482000	-2.533421000
Se	0.192982000	-1.583025000	-3.518648000
H	0.676278000	-4.499469000	-0.978688000
H	-0.036793000	-0.939175000	-0.235784000
H	-0.413707000	-3.104613000	-0.862491000
S	-1.017361000	-3.233300000	-4.959949000
C	0.369657000	-3.719451000	-6.024407000
H	1.053033000	-2.875899000	-6.143446000
H	0.909221000	-4.551574000	-5.569071000
H	-0.007351000	-4.035871000	-6.999855000
H	-1.269594000	-1.597713000	-5.702217000
O	-1.349340000	-0.577716000	-6.105296000
H	-0.970871000	-0.612521000	-6.992717000
H	-0.774089000	0.282342000	-5.468792000
O	-0.247164000	1.221625000	-4.913828000
H	0.347955000	0.983671000	-4.018092000
H	-0.930181000	1.839343000	-4.624369000

TS-SeH+SeS

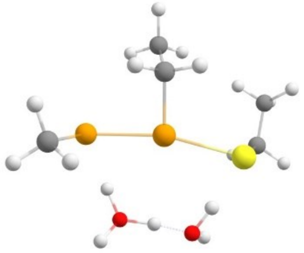

E= -3090.95
Nimag=-562

C	-0.038733000	0.928299000	-1.302855000
C	0.043493000	0.124871000	-0.019564000
C	0.146744000	-3.756083000	-1.514870000
C	1.063229000	-2.955016000	-2.411578000
H	0.110837000	1.994200000	-1.105766000
H	-1.019500000	0.799040000	-1.768242000
H	-0.720898000	0.455267000	0.691855000
H	1.025674000	0.238484000	0.445361000
H	-0.612276000	-4.270601000	-2.106148000
H	1.575741000	-3.589471000	-3.134397000
H	1.803727000	-2.384431000	-1.852299000
S	1.224238000	0.426851000	-2.515249000
Se	0.125383000	-1.580420000	-3.470711000
H	0.724404000	-4.507529000	-0.963770000
H	-0.115324000	-0.932465000	-0.231757000
H	-0.358708000	-3.113411000	-0.792368000
Se	-1.156831000	-3.332822000	-4.989128000
C	0.421208000	-3.762555000	-6.080357000
H	1.093440000	-2.904720000	-6.058246000
H	0.919281000	-4.632956000	-5.654327000
H	0.108307000	-3.983412000	-7.101441000
H	-1.332634000	-1.546529000	-5.740488000
O	-1.364803000	-0.527035000	-6.141110000
H	-0.958905000	-0.577452000	-7.016527000
H	-0.791820000	0.282458000	-5.506182000
O	-0.229600000	1.235977000	-4.925562000
H	0.339723000	0.989581000	-4.044587000
H	-0.889508000	1.881113000	-4.642772000

TS-SeH+SeSe

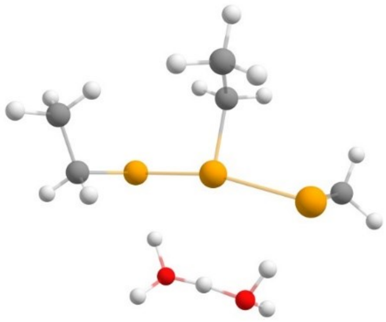

E= -3073.51
Nimag=-471

C	-0.033528000	0.469008000	-1.680468000
C	0.036237000	0.321279000	-0.177615000
C	0.070014000	-3.735707000	-1.368619000
C	1.165579000	-3.546814000	-2.396570000
H	-0.183682000	1.506288000	-1.978946000
H	-0.813964000	-0.146664000	-2.125867000
H	-0.898475000	0.667928000	0.278870000
H	0.857487000	0.915723000	0.226096000
H	-0.845164000	-4.105712000	-1.837013000
H	1.399781000	-4.484130000	-2.906443000
H	2.073390000	-3.152706000	-1.937033000
Se	1.613281000	-0.119430000	-2.595673000
Se	0.654979000	-2.250295000	-3.794991000
H	0.381225000	-4.455763000	-0.602879000
H	0.193684000	-0.720136000	0.107178000
H	-0.159078000	-2.789875000	-0.876532000
Se	3.050726000	1.913697000	-1.742854000
C	2.400583000	3.178513000	-3.101357000
H	3.195283000	3.883446000	-3.348617000
H	2.100682000	2.609791000	-3.981305000
H	1.542877000	3.718734000	-2.701656000
H	4.110492000	0.847095000	-3.038300000
O	4.621376000	0.255103000	-3.776929000
H	4.791965000	0.852946000	-4.516434000
H	4.019149000	-0.717159000	-4.180006000
O	3.538341000	-1.720280000	-4.656968000
H	2.512324000	-1.922914000	-4.400942000

H	4.038396000	-2.489966000	-4.355490000

TS-SH+SeSe

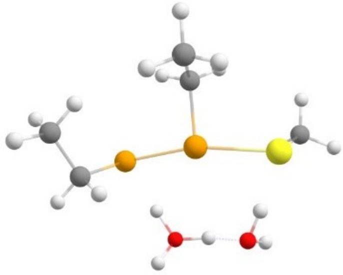

E=-565
Nimag= -3090.59

C	-0.027361000	0.479564000	-1.697148000
C	0.089472000	0.339044000	-0.196341000
C	0.063798000	-3.714546000	-1.379311000
C	1.177329000	-3.516006000	-2.386191000
H	-0.204196000	1.512908000	-1.995276000
H	-0.810578000	-0.149407000	-2.118403000
H	-0.834633000	0.677336000	0.287123000
H	0.916527000	0.943458000	0.179432000
H	-0.829195000	-4.121387000	-1.859836000
H	1.448485000	-4.457027000	-2.870070000
H	2.062867000	-3.086319000	-1.915173000
Se	1.600607000	-0.086260000	-2.654852000
Se	0.660890000	-2.260835000	-3.819830000
H	0.377744000	-4.408770000	-0.591232000
H	0.267057000	-0.699552000	0.087056000
H	-0.204425000	-2.765840000	-0.912976000
S	2.966494000	1.835659000	-1.865141000
C	2.405267000	3.086088000	-3.054977000
H	3.177182000	3.850486000	-3.172950000
H	2.198590000	2.613383000	-4.017255000
H	1.492485000	3.560333000	-2.690314000
H	4.038470000	0.872966000	-3.038333000
O	4.580820000	0.275458000	-3.751311000
H	4.784969000	0.864800000	-4.488027000
H	3.981122000	-0.737931000	-4.181704000
O	3.531531000	-1.718393000	-4.646749000
H	2.484230000	-1.932159000	-4.393643000
H	4.038413000	-2.475050000	-4.323931000

RX1 Model

RX1

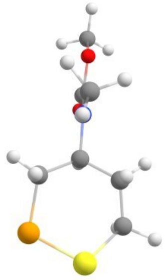

E= -3197.63
Nimag=0

C	-0.120893000	0.252037000	-0.650202000
C	0.708676000	-0.862806000	-0.033563000
C	0.330065000	-2.272721000	-0.482940000
C	0.541352000	-2.553674000	-1.963504000
H	0.154378000	1.213772000	-0.205464000
H	-1.189092000	0.088283000	-0.493737000
H	0.580144000	-0.829041000	1.053298000
H	1.762473000	-0.671309000	-0.247002000
H	-0.722459000	-2.435640000	-0.259782000
H	0.287489000	-3.593556000	-2.179897000
H	1.557925000	-2.345574000	-2.293978000
S	0.175004000	0.448777000	-2.428815000
Se	-0.658842000	-1.472011000	-3.076400000
N	1.047503000	-3.262637000	0.312330000
C	2.497512000	-3.258264000	0.331227000
H	2.890737000	-2.845888000	1.265786000
H	2.886628000	-4.269327000	0.209876000
H	2.853653000	-2.647776000	-0.495442000
C	0.319720000	-4.104694000	1.099796000
O	-0.898060000	-4.152275000	1.156379000
O	1.145721000	-4.909754000	1.833037000
C	0.446816000	-5.842574000	2.666910000
H	-0.189409000	-5.317322000	3.382481000
H	-0.175525000	-6.507229000	2.063626000
H	1.223383000	-6.406520000	3.183632000

RX1-H

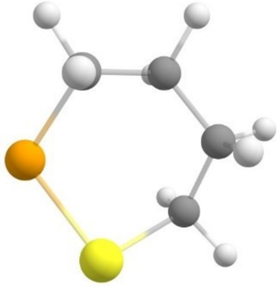

E= -1660.75
Nimag=0

C	-0.053919000	0.206596000	-0.574612000
C	0.416333000	-1.085826000	0.074092000
C	0.047431000	-2.361717000	-0.678101000
C	0.674685000	-2.500861000	-2.052774000
H	0.185470000	1.061506000	0.065913000
H	-1.131856000	0.195590000	-0.752459000
H	-0.022549000	-1.139691000	1.076969000
H	1.502493000	-1.038379000	0.198758000
H	-1.040765000	-2.424196000	-0.776782000
H	0.415755000	-3.458573000	-2.511844000
H	1.760112000	-2.397387000	-2.021468000
S	0.781820000	0.558930000	-2.147582000

Se	0.007786000	-1.139747000	-3.303432000
H	0.364317000	-3.219339000	-0.072198000

MeSSMe
E= -1095.48
Nimag=0

S	1.552541000	1.453761000	0.522412000
C	0.357745000	2.116089000	-0.671232000
H	-0.296838000	2.839230000	-0.184367000
H	0.871374000	2.574463000	-1.517092000
H	-0.228386000	1.261952000	-1.021898000
S	2.500878000	3.123082000	1.186455000
C	3.775857000	3.392062000	-0.075879000
H	4.357128000	4.256588000	0.256945000
H	4.422087000	2.517090000	-0.148276000
H	3.320870000	3.612045000	-1.042291000

RX1-H-SeH

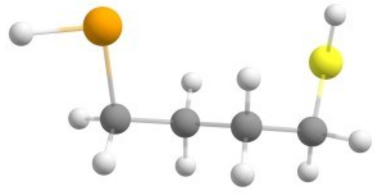

E= -1818.72
Nimag=0

C	-3.634152000	-0.497148000	0.055616000
C	-2.302078000	-1.225570000	0.104597000
C	-1.380246000	-0.737217000	1.208965000
C	-0.057819000	-1.473510000	1.274951000
H	-4.123979000	-0.525567000	1.033213000
H	-4.307896000	-0.966502000	-0.663468000
H	-1.808771000	-1.128230000	-0.866310000
H	-2.501039000	-2.295084000	0.245247000
H	-1.885880000	-0.866764000	2.173946000
H	-0.204732000	-2.550018000	1.372821000
H	0.546987000	-1.111028000	2.105117000
S	-3.514058000	1.278284000	-0.332512000
Se	0.987555000	-1.155133000	-0.376347000
H	-1.204568000	0.333273000	1.085407000
H	-2.989207000	1.112281000	-1.560503000
H	2.216926000	-1.748353000	0.172040000

RX1-H-SSe

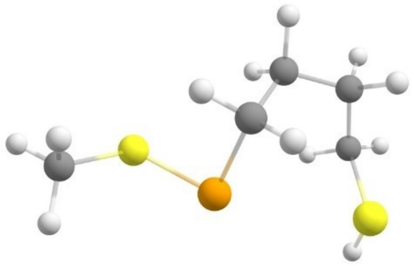

E= -2290.80
Nimag=0

C	-0.306786000	0.227380000	-0.551843000
C	0.472859000	-0.981439000	-0.046590000
C	0.279099000	-2.280783000	-0.820375000
C	0.658802000	-2.250319000	-2.287671000
H	-0.433607000	0.957083000	0.249133000
H	-1.282720000	-0.078231000	-0.929074000
H	0.165591000	-1.160100000	0.988850000
H	1.538945000	-0.731149000	-0.007567000
H	-0.759367000	-2.613727000	-0.735593000
H	0.804226000	-3.256847000	-2.676803000
H	1.561847000	-1.667414000	-2.472294000
S	0.605209000	1.036230000	-1.911328000
Se	-0.727030000	-1.474560000	-3.469167000
S	-1.924160000	-3.296310000	-3.752455000
C	-1.043936000	-4.139723000	-5.097160000
H	-0.027694000	-4.393104000	-4.792311000
H	-1.598824000	-5.059272000	-5.304654000
H	-1.023059000	-3.512877000	-5.988867000
H	0.890102000	-3.047453000	-0.327785000
H	-0.390267000	1.850436000	-2.304194000

RX1-H-SeSe

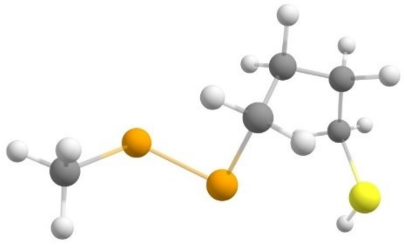

E= -2273.85
Nimag=0

C	-0.303204000	0.243819000	-0.525418000
C	0.468897000	-0.972557000	-0.026476000
C	0.277040000	-2.263310000	-0.814855000
C	0.663549000	-2.219609000	-2.279800000
H	-0.441348000	0.962359000	0.283755000
H	-1.273414000	-0.055904000	-0.921444000
H	0.153706000	-1.161181000	1.004841000
H	1.535412000	-0.726053000	0.023166000
H	-0.762159000	-2.596224000	-0.738551000
H	0.815133000	-3.222175000	-2.676144000
H	1.565403000	-1.631522000	-2.454265000
S	0.628391000	1.071012000	-1.860251000
Se	-0.711724000	-1.426598000	-3.462797000
Se	-2.032088000	-3.332047000	-3.733936000
C	-1.034830000	-4.221442000	-5.176317000
H	-0.027560000	-4.452227000	-4.833329000
H	-1.572778000	-5.144013000	-5.406149000
H	-1.007860000	-3.569381000	-6.047239000
H	0.884173000	-3.036236000	-0.327356000
H	-0.365510000	1.881112000	-2.265185000

RX1-H-SeS

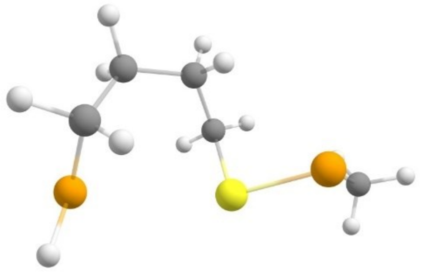

E= -2267.58
Nimag=0

C	0.031984000	0.165344000	-1.759632000
C	0.238421000	-0.628412000	-0.481046000

C	0.084593000	-2.141539000	-0.585389000
C	1.000138000	-2.844669000	-1.579960000
H	-0.230044000	1.199546000	-1.533820000
H	-0.763167000	-0.259134000	-2.376444000
H	-0.486771000	-0.272694000	0.259979000
H	1.227155000	-0.380514000	-0.084299000
H	-0.960102000	-2.390547000	-0.804705000
H	1.192610000	-3.872621000	-1.276201000
H	1.937907000	-2.307663000	-1.704261000
S	1.484992000	0.223730000	-2.866665000
Se	0.137312000	-2.927467000	-3.354732000
H	0.285503000	-2.561193000	0.406393000
Se	2.812329000	1.569079000	-1.767310000
C	2.085366000	3.298010000	-2.353845000
H	2.709122000	4.069750000	-1.897105000
H	2.141980000	3.353932000	-3.439279000
H	1.056985000	3.397285000	-2.010224000
H	1.375254000	-3.248982000	-4.080543000

RX1-H-SS

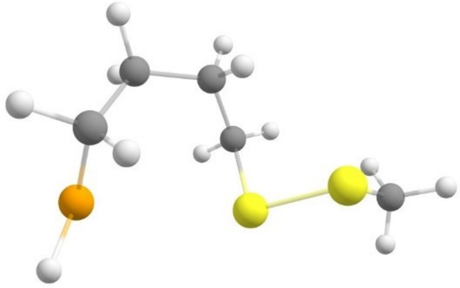

E= -2285.56
Nimag=0

C	0.001441000	0.190285000	-1.793286000
C	0.233992000	-0.584703000	-0.507693000
C	0.107437000	-2.101475000	-0.594266000
C	1.027057000	-2.797590000	-1.590417000
H	-0.288665000	1.219456000	-1.577367000
H	-0.780285000	-0.261168000	-2.407597000
H	-0.490558000	-0.233929000	0.236209000
H	1.221517000	-0.314076000	-0.123240000
H	-0.934428000	-2.372230000	-0.800826000
H	1.251113000	-3.814694000	-1.272314000
H	1.948843000	-2.239003000	-1.737064000
S	1.453702000	0.279586000	-2.898838000
Se	0.140516000	-2.934306000	-3.350186000
H	0.325533000	-2.506142000	0.400037000
S	2.697222000	1.503826000	-1.850920000
C	2.095407000	3.161050000	-2.278570000
H	2.757373000	3.870358000	-1.774028000
H	2.144557000	3.310211000	-3.357418000
H	1.074460000	3.303448000	-1.922084000
H	1.375335000	-3.237661000	-4.089221000

RX1-SSe

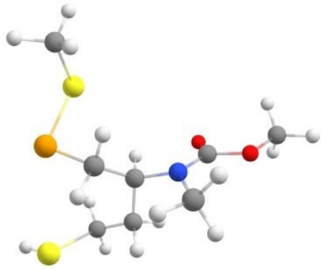

E= -3827.47
Nimag=0

C	0.375665000	-0.189105000	-1.029769000
C	1.037215000	-1.356118000	-0.310467000
C	1.074148000	-2.682725000	-1.067924000
C	1.786288000	-2.649748000	-2.410121000
H	0.022938000	0.543498000	-0.302755000
H	-0.461757000	-0.538162000	-1.633363000
H	0.478172000	-1.543445000	0.609747000
H	2.051833000	-1.075891000	-0.012004000
H	0.053147000	-3.017223000	-1.240191000
H	2.094389000	-3.654287000	-2.692240000
H	2.652164000	-1.989823000	-2.425633000
S	1.574435000	0.639403000	-2.129410000
Se	0.637474000	-2.015038000	-3.881112000
N	1.664840000	-3.708882000	-0.210536000
C	3.078850000	-3.639701000	0.104795000
H	3.258832000	-3.193371000	1.088448000
H	3.522408000	-4.635226000	0.093432000
H	3.571573000	-3.031109000	-0.650374000
C	0.811444000	-4.481642000	0.521704000
O	-0.404780000	-4.471814000	0.438698000
O	1.508292000	-5.296229000	1.371281000
C	0.673352000	-6.170524000	2.140162000
H	-0.025267000	-5.596394000	2.752697000
H	0.103907000	-6.833604000	1.484968000
H	1.355295000	-6.743651000	2.768439000
S	-0.552922000	-3.837388000	-4.126292000
C	0.530755000	-4.888207000	-5.132391000
H	0.755869000	-4.405885000	-6.083687000
H	1.453006000	-5.114535000	-4.595565000
H	-0.018908000	-5.817173000	-5.309298000
H	0.682525000	1.407372000	-2.779447000

RX1-SeSe

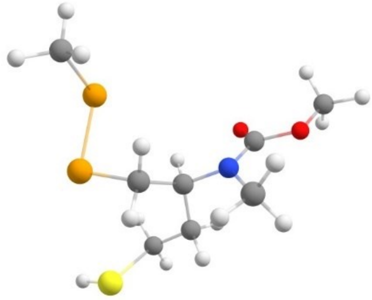

E= -3810.67
Nimag=0

C	0.383309000	-0.166494000	-0.996213000
C	1.045178000	-1.338318000	-0.284940000
C	1.071280000	-2.661966000	-1.047759000
C	1.775640000	-2.628158000	-2.394243000
H	0.034271000	0.563356000	-0.264627000
H	-0.456569000	-0.511309000	-1.598746000

H	0.491486000	-1.526882000	0.638259000
H	2.062915000	-1.063401000	0.007933000
H	0.047347000	-2.989807000	-1.215210000
H	2.081243000	-3.632458000	-2.678970000
H	2.641319000	-1.967882000	-2.412222000
S	1.579306000	0.664410000	-2.096793000
Se	0.621426000	-1.985154000	-3.858100000
N	1.659669000	-3.695125000	-0.197902000
C	3.077047000	-3.641857000	0.104570000
H	3.269716000	-3.211453000	1.092850000
H	3.512978000	-4.640473000	0.074312000
H	3.567099000	-3.025866000	-0.646280000
C	0.805691000	-4.467639000	0.533151000
O	-0.411168000	-4.447148000	0.459711000
O	1.502316000	-5.295744000	1.369875000
C	0.666239000	-6.169816000	2.137596000
H	-0.022021000	-5.595426000	2.761551000
H	0.085354000	-6.821648000	1.481152000
H	1.348339000	-6.754879000	2.754613000
Se	-0.654655000	-3.915095000	-4.111458000
C	0.566195000	-4.972943000	-5.230856000
H	0.762952000	-4.436366000	-6.156908000
H	1.489705000	-5.160349000	-4.685303000
H	0.056901000	-5.917067000	-5.437165000
H	0.684673000	1.426327000	-2.750087000

RX1-SeS

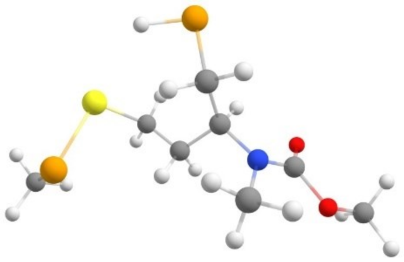

E= -3805.18
Nimag=0

C	0.075018000	-0.329825000	-1.780502000
C	0.802958000	-1.180194000	-0.756995000
C	1.034615000	-2.643669000	-1.121917000
C	1.785415000	-2.893211000	-2.429913000
H	-0.449707000	0.491477000	-1.290773000
H	-0.656407000	-0.913961000	-2.344042000
H	0.216986000	-1.181236000	0.166941000
H	1.752954000	-0.695667000	-0.522002000
H	0.071365000	-3.144809000	-1.179187000
H	2.322760000	-3.837700000	-2.356053000
H	2.486083000	-2.100260000	-2.677822000
S	1.124664000	0.407832000	-3.080485000
Se	0.618575000	-3.107579000	-4.000834000
N	1.733037000	-3.298354000	-0.018643000
C	3.084296000	-2.881273000	0.303075000
H	3.110249000	-2.197884000	1.157972000
H	3.706658000	-3.745701000	0.533735000
H	3.501926000	-2.372017000	-0.563022000
C	1.004488000	-4.085475000	0.821985000
O	-0.166944000	-4.392779000	0.673827000
O	1.771826000	-4.506917000	1.872680000
C	1.080016000	-5.379007000	2.774662000
H	0.208373000	-4.878661000	3.202120000
H	0.748920000	-6.283004000	2.258504000
H	1.803451000	-5.623856000	3.552425000
Se	2.554884000	1.610015000	-1.959521000
C	1.458394000	3.208883000	-1.644076000
H	2.102728000	3.928215000	-1.133687000
H	1.127988000	3.604216000	-2.602596000
H	0.611286000	2.950549000	-1.011114000
H	0.808236000	-1.722163000	-4.454742000

RX1-SS

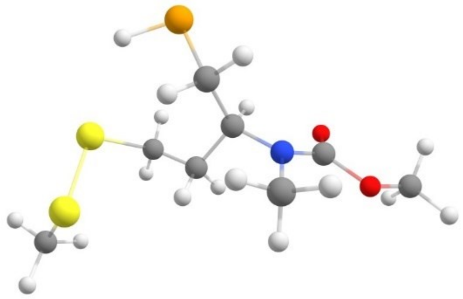

E= -3823.14
Nimag=0

C	0.033861000	-0.313481000	-1.783074000
C	0.764159000	-1.160515000	-0.758864000
C	1.014796000	-2.619467000	-1.129869000
C	1.766571000	-2.854506000	-2.440021000
H	-0.506533000	0.497902000	-1.293345000
H	-0.682119000	-0.900653000	-2.362351000
H	0.173368000	-1.172341000	0.161793000
H	1.707908000	-0.667479000	-0.517066000
H	0.058315000	-3.133493000	-1.186918000
H	2.313792000	-3.793631000	-2.371176000
H	2.459061000	-2.053491000	-2.684761000
S	1.097725000	0.447670000	-3.058750000
Se	0.598965000	-3.072917000	-4.010280000
N	1.724511000	-3.268078000	-0.030460000
C	3.072859000	-2.837577000	0.286717000
H	3.096046000	-2.165890000	1.150772000
H	3.708539000	-3.696669000	0.500947000
H	3.477086000	-2.310642000	-0.575028000
C	1.007868000	-4.064138000	0.811870000
O	-0.161142000	-4.382959000	0.668598000
O	1.784478000	-4.479401000	1.858087000
C	1.105549000	-5.358345000	2.763145000
H	0.231610000	-4.866278000	3.195516000
H	0.780104000	-6.265065000	2.248193000
H	1.835333000	-5.596895000	3.536918000
S	2.448584000	1.511339000	-1.982399000
C	1.550361000	3.039189000	-1.596747000
H	2.256497000	3.683269000	-1.065555000
H	1.225298000	3.524210000	-2.517214000
H	0.695926000	2.829250000	-0.952619000
H	0.795715000	-1.692934000	-4.476053000

TS-RX1-H-SeS

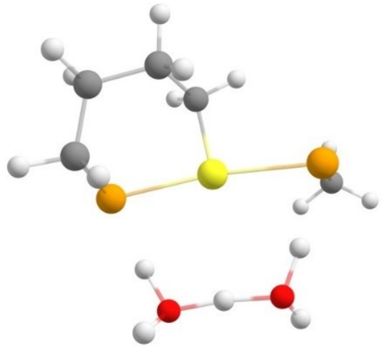

E= -2915.10

Nimag=-481

C	0.070433000	0.082810000	-1.284488000
C	0.224736000	-1.007371000	-0.238431000
C	-0.051808000	-2.428145000	-0.716143000
C	0.868957000	-2.935631000	-1.810401000
H	0.116209000	1.065016000	-0.815674000
H	-0.876176000	-0.012475000	-1.818412000
H	-0.469954000	-0.784834000	0.580653000
H	1.235627000	-0.948165000	0.176566000
H	-1.087851000	-2.500446000	-1.062881000
H	0.726829000	-4.005465000	-1.974004000
H	1.913500000	-2.743819000	-1.559189000
S	1.397526000	0.092894000	-2.536514000
Se	0.519538000	-2.045310000	-3.531308000
H	0.040131000	-3.101161000	0.145723000
Se	2.693765000	2.058367000	-1.608902000
C	2.087020000	3.333368000	-2.974673000
H	2.863991000	4.080697000	-3.141081000
H	1.882388000	2.780046000	-3.890675000
H	1.174924000	3.817763000	-2.626756000
H	3.870499000	0.992498000	-2.784399000
O	4.443817000	0.368557000	-3.443326000
H	4.682329000	0.926625000	-4.194722000
H	3.876792000	-0.636521000	-3.837131000
O	3.433740000	-1.653927000	-4.297036000
H	2.400836000	-1.841508000	-4.052320000
H	3.928634000	-2.407584000	-3.950349000

RX1-H-TS-SS

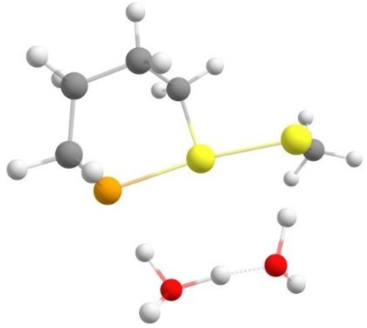

E= -2932.27

Nimag=-570

C	0.083563000	0.113804000	-1.338136000
C	0.233516000	-0.954771000	-0.269416000
C	-0.037396000	-2.385705000	-0.719410000
C	0.890199000	-2.914433000	-1.798127000
H	0.129069000	1.105401000	-0.888880000
H	-0.860665000	0.008605000	-1.874027000
H	-0.466480000	-0.716611000	0.540728000
H	1.241702000	-0.884631000	0.150196000
H	-1.071722000	-2.468008000	-1.069126000
H	0.760795000	-3.990179000	-1.929358000
H	1.932005000	-2.703187000	-1.551100000
S	1.414661000	0.098028000	-2.583595000
Se	0.533318000	-2.077324000	-3.544539000
H	0.051752000	-3.039543000	0.157366000
S	2.639615000	1.927944000	-1.719663000
C	2.091129000	3.215229000	-2.871792000
H	2.836009000	4.014114000	-2.907210000
H	1.959053000	2.786039000	-3.866794000
H	1.138702000	3.629372000	-2.535806000
H	3.807958000	1.006206000	-2.806109000
O	4.408050000	0.391765000	-3.457276000
H	4.645078000	0.947650000	-4.209836000
H	3.850572000	-0.650562000	-3.858563000
O	3.431754000	-1.657102000	-4.301528000
H	2.385732000	-1.859237000	-4.052368000
H	3.938467000	-2.396582000	-3.941503000

RX1-TS-SS

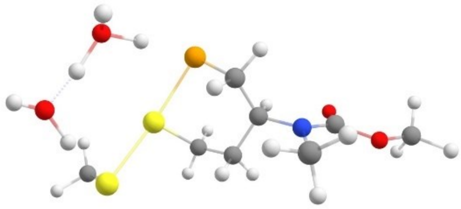

E= -4469.35

Nimag=-564

C	0.326940000	-0.054217000	-1.152995000
C	1.115356000	-1.098467000	-0.385328000
C	1.009610000	-2.525784000	-0.915042000
C	1.520627000	-2.727658000	-2.334929000
H	0.326213000	0.887879000	-0.605615000
H	-0.702892000	-0.373555000	-1.313304000
H	0.745593000	-1.116903000	0.645610000
H	2.161538000	-0.786616000	-0.350949000
H	-0.035167000	-2.829103000	-0.887586000
H	1.588624000	-3.795087000	-2.545960000
H	2.492278000	-2.257757000	-2.484374000
S	1.033011000	0.336394000	-2.785310000
Se	0.296333000	-1.931684000	-3.649291000
N	1.692825000	-3.441380000	-0.003775000
C	3.116134000	-3.289564000	0.224176000
H	3.327005000	-2.830016000	1.195054000
H	3.618598000	-4.256646000	0.184710000
H	3.523902000	-2.652667000	-0.557500000
C	0.937380000	-4.301074000	0.733739000
O	-0.271412000	-4.444450000	0.650181000
O	1.724988000	-5.010170000	1.601278000
C	0.997332000	-5.959282000	2.388961000
H	0.238412000	-5.457890000	2.993697000
H	0.506054000	-6.695993000	1.749155000
H	1.741360000	-6.438608000	3.025524000
S	2.115431000	2.334114000	-2.158849000
C	0.916051000	3.533245000	-2.798333000
H	1.413785000	4.492022000	-2.963040000
H	0.494215000	3.167496000	-3.736257000
H	0.109435000	3.668296000	-2.075575000

H	2.915951000	1.798448000	-3.732532000
O	3.318062000	1.389931000	-4.644251000
H	3.149184000	2.045923000	-5.331910000
H	2.841791000	0.289313000	-4.981642000
O	2.459781000	-0.743469000	-5.401761000
H	1.684017000	-1.220055000	-4.799449000
H	3.194464000	-1.369494000	-5.438576000

RX1-TS-SeS

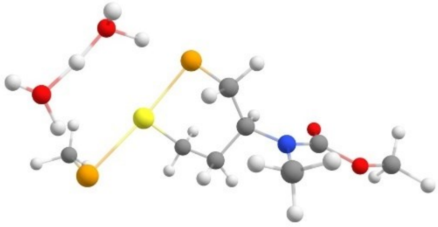

E= -4452.17
Nimag=-477

C	0.337834000	-0.063194000	-1.109441000
C	1.126981000	-1.114667000	-0.352278000
C	1.010113000	-2.538362000	-0.888807000
C	1.510574000	-2.735466000	-2.312873000
H	0.340726000	0.874907000	-0.555756000
H	-0.693473000	-0.379706000	-1.267109000
H	0.764660000	-1.136949000	0.681242000
H	2.174915000	-0.807912000	-0.323832000
H	-0.036385000	-2.835275000	-0.857082000
H	1.556723000	-3.801886000	-2.535321000
H	2.490179000	-2.282805000	-2.463225000
S	1.038493000	0.336161000	-2.743669000
Se	0.294531000	-1.906090000	-3.613536000
N	1.693067000	-3.464301000	0.011903000
C	3.117826000	-3.321101000	0.236289000
H	3.334128000	-2.879458000	1.214190000
H	3.617291000	-4.288865000	0.177659000
H	3.524400000	-2.671671000	-0.535625000
C	0.937505000	-4.332480000	0.739419000
O	-0.271610000	-4.472921000	0.655906000
O	1.725491000	-5.053568000	1.596489000
C	0.997263000	-6.009141000	2.375890000
H	0.242848000	-5.511846000	2.989586000
H	0.500461000	-6.736271000	1.729464000
H	1.741957000	-6.498780000	3.003745000
Se	2.176656000	2.486176000	-2.080021000
C	0.838338000	3.665929000	-2.901872000
H	1.319234000	4.595169000	-3.210108000
H	0.410026000	3.150650000	-3.760894000
H	0.056932000	3.876385000	-2.172147000
H	2.978430000	1.810000000	-3.756443000
O	3.357161000	1.380336000	-4.663669000
H	3.184658000	2.030680000	-5.356796000
H	2.874396000	0.307227000	-4.974031000
O	2.471098000	-0.746424000	-5.392376000
H	1.704273000	-1.204643000	-4.792765000
H	3.195109000	-1.384366000	-5.434151000

RX1-TS-SeSe

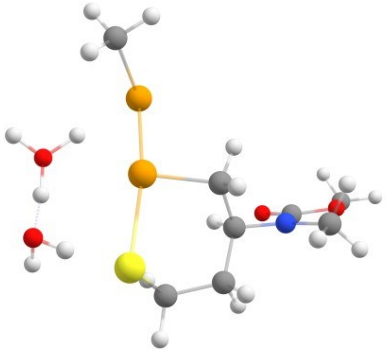

E= -4456.93
Nimag=-532

C	0.528553000	-0.047840000	-1.068796000
C	1.274471000	-1.080733000	-0.239161000
C	1.118237000	-2.523323000	-0.713375000
C	1.695423000	-2.825030000	-2.087481000
H	0.506780000	0.905350000	-0.533669000
H	-0.500425000	-0.378440000	-1.233715000
H	0.891584000	-1.044427000	0.785738000
H	2.331281000	-0.804836000	-0.203958000
H	0.057768000	-2.764729000	-0.734456000
H	1.640979000	-3.893563000	-2.287200000
H	2.720725000	-2.476583000	-2.196016000
S	1.302109000	0.256435000	-2.685692000
Se	0.662741000	-1.990492000	-3.528273000
N	1.694838000	-3.435950000	0.270420000
C	3.132141000	-3.477487000	0.453881000
H	3.386453000	-3.466009000	1.514395000
H	3.574365000	-4.370899000	0.002086000
H	3.565958000	-2.599026000	-0.018247000
C	0.864737000	-4.315245000	0.896064000
O	-0.349551000	-4.356948000	0.782392000
O	1.579884000	-5.164503000	1.696857000
C	0.764509000	-6.101956000	2.409054000
H	0.062244000	-5.582616000	3.065283000
H	0.199502000	-6.726989000	1.714038000
H	1.459780000	-6.706280000	2.992059000
Se	-0.550128000	-4.140283000	-4.434574000
C	0.454335000	-4.270718000	-6.121373000
H	0.770167000	-3.271666000	-6.419648000
H	1.329847000	-4.899158000	-5.960447000
H	-0.185839000	-4.714077000	-6.885152000
H	-1.571578000	-2.523450000	-4.874430000
O	-2.081560000	-1.584963000	-5.089360000
H	-2.108277000	-1.505945000	-6.051843000
H	-1.605212000	-0.607392000	-4.625293000
O	-1.204319000	0.496644000	-4.208405000
H	-0.288658000	0.469375000	-3.652631000
H	-1.863115000	0.876442000	-3.613667000

RX1-TS-SSe

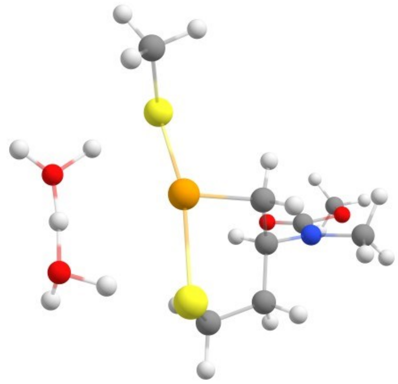

E= -4474.19
Nimag=-695

C	0.501815000	-0.049195000	-1.076948000
C	1.256194000	-1.082725000	-0.255429000
C	1.108189000	-2.524386000	-0.735966000
C	1.683064000	-2.819328000	-2.112608000
H	0.462817000	0.896354000	-0.529703000
H	-0.520966000	-0.392162000	-1.254435000
H	0.874913000	-1.055189000	0.770270000
H	2.311194000	-0.800027000	-0.219669000
H	0.049244000	-2.772689000	-0.754669000
H	1.619389000	-3.886108000	-2.320205000
H	2.711659000	-2.479730000	-2.217632000
S	1.283276000	0.283604000	-2.684977000
Se	0.657939000	-1.971227000	-3.549104000
N	1.694656000	-3.436954000	0.242263000
C	3.132985000	-3.470291000	0.419012000
H	3.391194000	-3.471174000	1.478539000
H	3.580337000	-4.354184000	-0.046440000
H	3.558524000	-2.582192000	-0.042587000
C	0.872932000	-4.324574000	0.867619000
O	-0.341671000	-4.371453000	0.761590000
O	1.597635000	-5.175645000	1.657964000
C	0.791532000	-6.122185000	2.368740000
H	0.090327000	-5.611081000	3.032500000
H	0.225813000	-6.745603000	1.672841000
H	1.493491000	-6.726616000	2.943569000
S	-0.506372000	-3.990791000	-4.404810000
C	0.405953000	-4.259038000	-5.951839000
H	0.693783000	-3.301426000	-6.389584000
H	1.307223000	-4.840259000	-5.749485000
H	-0.224870000	-4.810349000	-6.653514000
H	-1.509664000	-2.528651000	-4.873339000
O	-2.034737000	-1.599433000	-5.100846000
H	-2.064417000	-1.529007000	-6.063066000
H	-1.565718000	-0.577007000	-4.624900000
O	-1.193437000	0.496325000	-4.217717000
H	-0.265950000	0.476713000	-3.639895000
H	-1.867524000	0.860375000	-3.630133000

RX1-H-TS-SeSe

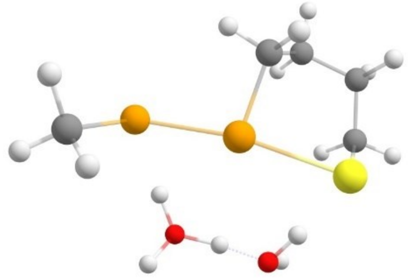

E= -2919.80
Nimag=-557

C	0.124783000	0.122593000	-1.183537000
C	0.218741000	-1.007172000	-0.168290000
C	-0.165058000	-2.388822000	-0.687744000
C	0.723597000	-2.954657000	-1.777452000
H	0.207862000	1.087249000	-0.675516000
H	-0.838305000	0.081563000	-1.700192000
H	-0.445303000	-0.763810000	0.669511000
H	1.237796000	-1.035837000	0.230466000
H	-1.197828000	-2.369429000	-1.049509000
H	0.449366000	-3.980380000	-2.021181000
H	1.778607000	-2.912113000	-1.505646000
S	1.448284000	0.060087000	-2.430202000
Se	0.549815000	-1.993228000	-3.482315000
H	-0.138395000	-3.086607000	0.159027000
Se	-0.787204000	-3.809590000	-4.862482000
C	0.706613000	-4.385538000	-6.006461000
H	1.405869000	-3.556421000	-6.108826000
H	1.206175000	-5.229727000	-5.531889000
H	0.318991000	-4.683987000	-6.981364000
H	-1.020744000	-2.029797000	-5.635057000
O	-1.105161000	-1.010422000	-6.021073000
H	-0.698386000	-1.020722000	-6.897313000
H	-0.608226000	-0.158761000	-5.370179000
O	-0.122639000	0.832696000	-4.789453000
H	0.460120000	0.610991000	-3.915011000
H	-0.833597000	1.414493000	-4.493173000

RX1-H-TS-SSe

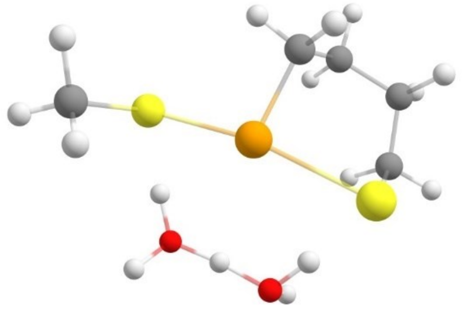

E= -2937.22
Nimag=-731

C	0.083545000	0.152489000	-1.163864000
C	0.187094000	-0.988843000	-0.162257000
C	-0.155662000	-2.372022000	-0.706058000
C	0.767670000	-2.906813000	-1.782692000
H	0.121332000	1.111696000	-0.640676000
H	-0.863542000	0.087144000	-1.706853000
H	-0.497464000	-0.772512000	0.666302000
H	1.198995000	-0.998889000	0.255287000
H	-1.180395000	-2.369597000	-1.090225000
H	0.514173000	-3.932348000	-2.049508000

H	1.815061000	-2.854500000	-1.484326000
S	1.439996000	0.149060000	-2.376986000
Se	0.618021000	-1.925139000	-3.476355000
H	-0.132572000	-3.079177000	0.132987000
S	-0.610694000	-3.639366000	-4.816824000
C	0.724796000	-4.230900000	-5.896198000
H	1.413825000	-3.414091000	-6.119067000
H	1.274626000	-5.029379000	-5.395011000
H	0.298953000	-4.618590000	-6.824997000
H	-0.921127000	-2.007845000	-5.574716000
O	-1.052371000	-0.990072000	-5.957916000
H	-0.674704000	-0.982340000	-6.846179000
H	-0.558851000	-0.089969000	-5.295983000
O	-0.109927000	0.879767000	-4.734836000
H	0.480336000	0.665506000	-3.835206000
H	-0.839754000	1.443192000	-4.448832000

RX1-TS-selenol-generation

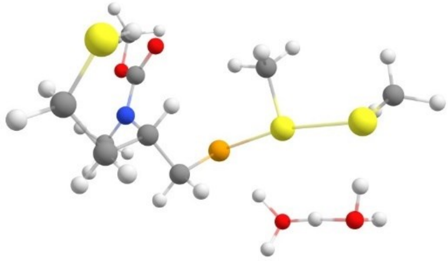

E= -5105.71
Nimag=-641

C	-3.106520000	-1.519797000	-0.781859000
C	-1.767619000	-1.790057000	-1.445575000
C	-0.713565000	-0.737570000	-1.121049000
C	0.632683000	-1.089892000	-1.727328000
H	-3.832238000	-2.280227000	-1.078220000
H	-3.497538000	-0.548562000	-1.087309000
H	-1.914629000	-1.851801000	-2.529957000
H	-1.387364000	-2.759042000	-1.114777000
H	-0.595223000	-0.703377000	-0.042965000
H	0.592062000	-1.100840000	-2.817216000
H	0.930413000	-2.074978000	-1.369863000
S	-3.069344000	-1.586741000	1.035916000
Se	2.031543000	0.165059000	-1.159353000
N	-1.147243000	0.596788000	-1.516259000
C	-1.141534000	0.932956000	-2.924614000
H	-0.174784000	1.341571000	-3.234639000
H	-1.341960000	0.028388000	-3.496903000
H	-1.920655000	1.660875000	-3.140363000
C	-1.288292000	1.545731000	-0.555870000
O	-1.275536000	1.345913000	0.651702000
O	-1.477393000	2.779881000	-1.103026000
C	-1.674520000	3.817293000	-0.134831000
H	-0.799243000	3.908396000	0.511791000
H	-1.820067000	4.728669000	-0.714747000
H	-2.550976000	3.608451000	0.482575000
S	2.417021000	-1.238647000	1.000234000
C	1.313782000	-0.318341000	2.109890000
H	0.544026000	-0.989328000	2.488082000
H	1.889381000	0.097939000	2.936392000
H	0.853713000	0.478799000	1.534020000
H	-2.391098000	-0.433522000	1.194608000
S	3.005257000	-2.799672000	2.613877000
C	4.240634000	-1.839824000	3.527411000

H	4.929049000	-2.522038000	4.031615000
H	4.787475000	-1.199237000	2.832705000
H	3.745179000	-1.218688000	4.274887000
H	4.045165000	-3.228020000	1.203113000
O	4.636355000	-3.389239000	0.296323000
H	5.560304000	-3.280664000	0.554451000
H	4.385337000	-2.611127000	-0.599276000
O	4.221088000	-1.891806000	-1.555381000
H	3.457739000	-1.126130000	-1.444891000
H	3.951153000	-2.420786000	-2.316932000

RX1-TS-thiol-echange

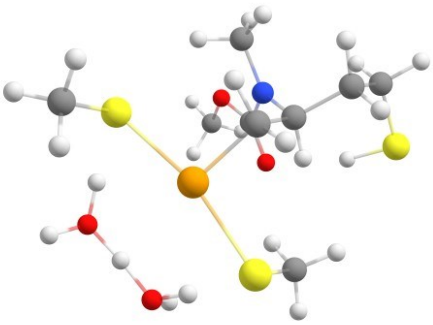

E= -5112.61
Nimag=-744

C	-3.023470000	-1.425749000	-0.933908000
C	-1.704865000	-1.732521000	-1.621682000
C	-0.632285000	-0.688737000	-1.332729000
C	0.689014000	-1.028156000	-2.000190000
H	-3.761411000	-2.193555000	-1.175843000
H	-3.417843000	-0.465778000	-1.268455000
H	-1.877045000	-1.819418000	-2.699940000
H	-1.329304000	-2.698609000	-1.277243000
H	-0.473174000	-0.673255000	-0.260916000
H	0.675573000	-0.831484000	-3.070715000
H	0.951318000	-2.069957000	-1.826768000
S	-2.935958000	-1.417909000	0.883611000
Se	2.167142000	0.024550000	-1.255446000
N	-1.070355000	0.654279000	-1.677779000
C	-1.311964000	0.983530000	-3.069825000
H	-0.475884000	1.537813000	-3.498244000
H	-1.440475000	0.055562000	-3.622819000
H	-2.223673000	1.573372000	-3.166203000
C	-1.066061000	1.609766000	-0.714615000
O	-0.897120000	1.420394000	0.483693000
O	-1.305421000	2.839422000	-1.250057000
C	-1.325397000	3.899179000	-0.287977000
H	-0.356119000	3.982327000	0.208845000
H	-1.540642000	4.802509000	-0.858545000
H	-2.096763000	3.724792000	0.465317000
S	2.538357000	-1.451534000	0.684542000
C	1.210942000	-0.965127000	1.827920000
H	1.596631000	-0.995521000	2.849719000
H	0.860443000	0.037362000	1.584695000
H	0.374933000	-1.659275000	1.744899000
H	-2.208418000	-0.288765000	0.985430000
S	2.229027000	1.883556000	-2.970319000
C	3.524454000	1.227544000	-4.059799000
H	4.232372000	0.634461000	-3.477526000
H	3.075873000	0.589376000	-4.823429000
H	4.046359000	2.052816000	-4.550417000
H	3.303858000	2.367070000	-1.599520000

O	3.954278000	2.576838000	-0.736404000
H	4.851448000	2.655432000	-1.083464000
H	3.939509000	1.760023000	0.168020000
O	3.988363000	1.032586000	1.130841000
H	3.507474000	0.056722000	0.984605000
H	3.486018000	1.454831000	1.839802000

RX1-H-TS-selenol-generation

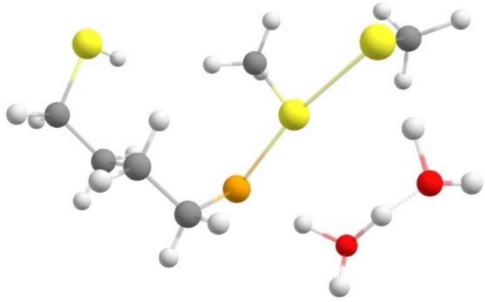

E= -3563.74
Nimag=-609

C	-3.667734000	-0.539264000	0.034968000
C	-2.355816000	-1.302141000	0.085264000
C	-1.417096000	-0.833519000	1.180939000
C	-0.085247000	-1.554177000	1.191878000
H	-4.141835000	-0.517866000	1.020502000
H	-4.369270000	-1.008748000	-0.657084000
H	-1.852132000	-1.222882000	-0.881369000
H	-2.585662000	-2.364776000	0.233960000
H	-1.901125000	-0.985746000	2.154724000
H	-0.214366000	-2.637516000	1.255924000
H	0.519221000	-1.215304000	2.033716000
S	-3.502286000	1.217977000	-0.417774000
Se	0.989531000	-1.201832000	-0.426467000
S	1.609215000	1.136551000	0.382767000
C	0.483770000	1.975573000	-0.773264000
H	0.540881000	3.041285000	-0.557831000
H	0.798454000	1.781630000	-1.798524000
H	-0.532777000	1.623206000	-0.619798000
H	-1.240102000	0.237662000	1.082900000
H	-3.023140000	0.992484000	-1.654415000
S	2.510717000	3.085633000	1.370464000
C	3.636878000	3.553604000	0.030249000
H	4.421715000	4.205488000	0.421622000
H	4.078310000	2.654746000	-0.405518000
H	3.086093000	4.088647000	-0.745391000
H	3.535402000	1.702029000	1.999569000
O	4.104699000	0.826059000	2.279516000
H	5.024643000	1.035848000	2.076133000
H	3.773748000	-0.216426000	1.680121000
O	3.545550000	-1.253877000	1.172475000
H	3.410578000	-1.920196000	1.858672000
H	2.639291000	-1.278920000	0.551371000

RX1-H-TS-thiol-exchange

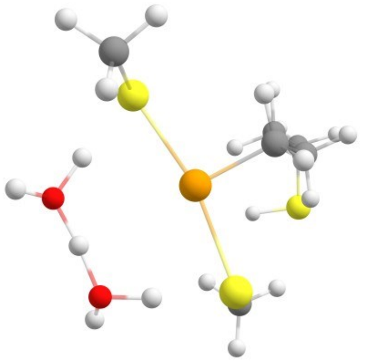

E= -3570.27
Nimag=-788

C	-3.750614000	-0.848971000	0.212717000
C	-2.364536000	-1.467280000	0.206159000
C	-1.463880000	-0.967864000	1.319037000
C	-0.056690000	-1.520334000	1.250986000
H	-4.225675000	-0.965650000	1.191179000
H	-4.395013000	-1.329232000	-0.526056000
H	-1.883904000	-1.274143000	-0.755892000
H	-2.474017000	-2.555256000	0.284192000
H	-1.896971000	-1.248361000	2.288380000
H	-0.043759000	-2.609386000	1.202591000
H	0.546103000	-1.189462000	2.095461000
S	-3.776652000	0.950023000	-0.080996000
Se	0.941990000	-0.949951000	-0.346978000
S	1.448634000	1.339653000	0.460262000
C	0.091200000	2.247801000	-0.334028000
H	0.390478000	3.289187000	-0.474603000
H	-0.135798000	1.793076000	-1.300206000
H	-0.802287000	2.208761000	0.286915000
H	-1.426550000	0.121209000	1.295833000
H	-3.165389000	0.897278000	-1.278090000
S	0.770004000	-3.102288000	-1.627840000
C	2.119752000	-4.011685000	-0.823645000
H	2.918357000	-3.319497000	-0.549188000
H	1.749083000	-4.497641000	0.080655000
H	2.504795000	-4.776358000	-1.502628000
H	1.833332000	-1.989391000	-2.580133000
O	2.498614000	-1.264703000	-3.076764000
H	3.351246000	-1.708031000	-3.168210000
H	2.665943000	-0.207571000	-2.495901000
O	2.902159000	0.874178000	-2.009789000
H	2.622537000	1.563688000	-2.624692000
H	2.398625000	1.095262000	-1.051061000

RX1-TS-selenol-generation-inter.

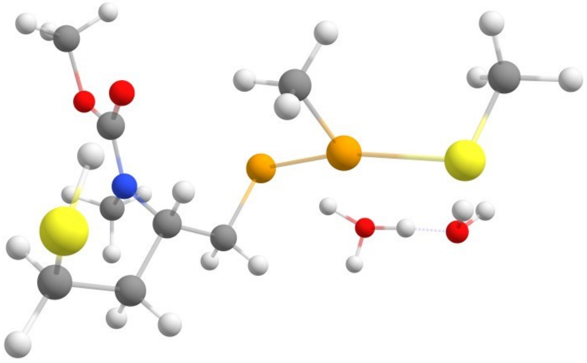

E= -5093.86

Nimag=-601

C	-4.648063000	-3.311357000	5.920544000
C	-3.191852000	-2.880591000	5.907507000
C	-2.980515000	-1.498805000	5.299262000
C	-1.528014000	-1.067439000	5.384250000
H	-4.736563000	-4.320481000	6.328633000
H	-5.054086000	-3.324204000	4.908387000
H	-2.605571000	-3.626144000	5.358277000
H	-2.810628000	-2.858577000	6.930789000
H	-3.572530000	-0.788266000	5.867365000
H	-0.879966000	-1.717299000	4.794940000
H	-1.213998000	-1.096800000	6.426861000
S	-5.724353000	-2.283806000	6.967081000
Se	-1.292380000	0.783772000	4.765226000
N	-3.479633000	-1.430336000	3.931485000
C	-2.716576000	-2.081026000	2.886958000
H	-1.964214000	-1.409189000	2.462371000
H	-2.214714000	-2.947966000	3.314097000
H	-3.379078000	-2.418816000	2.092962000
C	-4.482714000	-0.560755000	3.647138000
O	-5.156146000	0.044886000	4.470476000
O	-4.678897000	-0.453692000	2.302848000
C	-5.754310000	0.422319000	1.943253000
H	-5.555858000	1.436259000	2.296812000
H	-5.795090000	0.396234000	0.854370000
H	-6.696136000	0.074747000	2.373409000
H	-5.712920000	-1.211045000	6.152369000
C	-1.906327000	4.297974000	9.270420000
C	-3.864693000	1.936371000	6.983299000
H	-1.506592000	4.793243000	10.158657000
H	-1.302960000	4.552205000	8.397009000
H	-4.110424000	1.600876000	5.980104000
H	-4.350963000	1.306492000	7.724859000
S	-1.891973000	2.501441000	9.527246000
Se	-1.925087000	1.751846000	7.191733000
H	0.309735000	1.049698000	5.828566000
O	1.179280000	1.180529000	6.482057000
H	1.589131000	0.308632000	6.551778000
H	0.965414000	1.597509000	7.572751000
O	0.877107000	2.026456000	8.730514000
H	-0.130143000	2.205981000	9.087171000
H	1.334849000	2.874308000	8.790108000
H	-4.133735000	2.981694000	7.121743000
H	-2.930444000	4.636404000	9.105886000

RX1-TS-selenol-generation-intra.

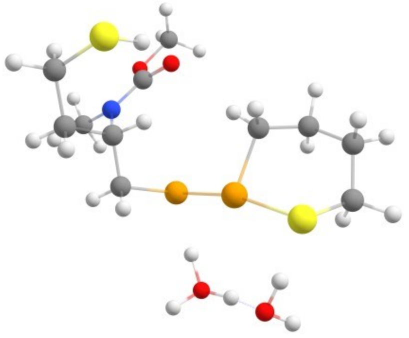

E= -5678.22
Nimag=-546

C	-4.666034000	-3.360671000	5.895067000
C	-3.194791000	-2.985691000	5.864042000
C	-2.941384000	-1.586524000	5.314338000
C	-1.467892000	-1.225766000	5.363346000
H	-4.783432000	-4.384670000	6.256259000
H	-5.096488000	-3.308892000	4.894274000
H	-2.652747000	-3.727049000	5.265718000
H	-2.787755000	-3.026661000	6.876718000
H	-3.476191000	-0.875601000	5.937561000
H	-0.870677000	-1.873519000	4.720803000
H	-1.117950000	-1.320943000	6.390207000
S	-5.676326000	-2.347436000	7.018235000
Se	-1.180997000	0.641225000	4.816942000
N	-3.484184000	-1.430008000	3.970417000
C	-2.787402000	-2.062283000	2.869727000
H	-2.020033000	-1.404286000	2.450426000
H	-2.311427000	-2.969824000	3.237992000
H	-3.490980000	-2.332112000	2.085115000
C	-4.454218000	-0.503781000	3.763066000
O	-5.074588000	0.088537000	4.636321000
O	-4.685883000	-0.318758000	2.432311000
C	-5.729403000	0.621957000	2.152769000
H	-5.472705000	1.606852000	2.548771000
H	-5.805552000	0.653417000	1.065905000
H	-6.672794000	0.296519000	2.596661000
H	-5.628896000	-1.231306000	6.265364000
C	-2.264890000	4.113080000	8.992599000
C	-3.655091000	4.248718000	8.387951000
C	-3.836804000	3.614850000	7.012543000
C	-3.680630000	2.109592000	6.952336000
H	-2.174685000	4.757503000	9.871375000
H	-1.506401000	4.407684000	8.262008000
H	-3.881627000	5.318183000	8.304146000
H	-4.382745000	3.822126000	9.085663000
H	-3.138784000	4.072595000	6.304316000
H	-3.934192000	1.713548000	5.972879000
H	-4.286885000	1.605218000	7.704905000
S	-1.905210000	2.415467000	9.539599000
Se	-1.833253000	1.526650000	7.261576000
H	-4.848427000	3.855272000	6.661416000
H	0.406489000	0.974963000	5.912475000
O	1.255933000	1.181788000	6.564288000
H	1.703566000	0.336542000	6.699317000
H	1.016589000	1.675578000	7.616230000
O	0.911058000	2.187586000	8.742168000
H	-0.098682000	2.306381000	9.089742000
H	1.290087000	3.075332000	8.724205000

MeSeSMe
E= -1077.45
Nimag=0

C	-1.862672000	4.303482000	9.224232000
C	-3.915068000	1.924640000	7.033664000
H	-1.752991000	4.673394000	10.247946000
H	-1.039991000	4.670278000	8.610535000
H	-4.120729000	1.567511000	6.021857000
H	-4.364887000	1.261552000	7.770138000
S	-1.811036000	2.492859000	9.335495000
Se	-1.962226000	1.881821000	7.247019000
H	-4.275152000	2.945339000	7.150416000
H	-2.818446000	4.637671000	8.819654000

RX1-SSe-crest

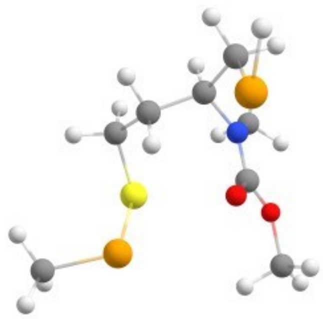

E= -3804.74
Nimag=0

C	0.992848000	-0.134466000	2.180337000
C	-0.364019000	0.331541000	1.690529000
C	-1.355851000	-0.782778000	1.381975000
C	-2.788302000	-0.285242000	1.226392000
H	0.902220000	-0.950350000	2.906620000
H	1.519028000	0.686501000	2.670146000
H	-0.239195000	0.978448000	0.826651000
H	-0.818978000	0.941649000	2.477246000
H	-1.384346000	-1.420271000	2.270452000
H	-3.116200000	0.131803000	2.178123000
H	-3.431934000	-1.122976000	0.959539000
S	2.102786000	-0.807933000	0.897785000
Se	-3.050269000	1.147137000	-0.112606000
N	-0.960070000	-1.701260000	0.312337000
C	-0.768788000	-3.091302000	0.674183000
H	0.221295000	-3.257588000	1.115143000
H	-1.535577000	-3.374392000	1.397685000
H	-0.867639000	-3.723682000	-0.204073000
C	-0.546582000	-1.208995000	-0.883642000
O	-0.626174000	-0.039386000	-1.225163000
O	-0.037046000	-2.186674000	-1.682833000
C	0.424977000	-1.705435000	-2.951891000
H	0.771139000	-2.590291000	-3.485839000
H	-0.384823000	-1.216152000	-3.496898000
H	1.242483000	-0.996351000	-2.812238000
H	-4.306676000	1.571201000	0.542973000
Se	2.408464000	0.911582000	-0.403458000
C	4.001146000	1.672243000	0.463489000
H	4.279847000	2.550011000	-0.124838000
H	3.755188000	1.969741000	1.481642000
H	4.805244000	0.938904000	0.454007000

[1] B. Thapa, H. Bernhard Schlegel, *J. Phys. Chem. A* **2016**, *120*, 8916–8922.
[2] M. Bortoli, L. P. Wolters, L. Orian, F. M. Bickelhaupt, *J. Chem. Theory Comput.* **2016**, *12*, 2752–2761.
